# Consequences of *Shigella* infection in young children: a systematic review

**DOI:** 10.1016/j.ijid.2023.01.034

**Published:** 2023-04

**Authors:** Tanya E. Libby, Miranda L.M. Delawalla, Fatima Al-Shimari, Calman A. MacLennan, Kirsten S. Vannice, Patricia B. Pavlinac

**Affiliations:** 1 Department of Epidemiology, University of Washington, Seattle, Washington, USA; 2 Department of Global Health, University of Washington, Seattle, Washington, USA; 3 Bill and Melinda Gates Foundation, London, UK; 4 Bill and Melinda Gates Foundation, Seattle, Washington, USA

**Keywords:** Shigella, Stunting, Enteric, Diarrhea, Growth faltering, Vaccine

## Abstract

**Objectives:**

We conducted a systematic review of the longitudinal consequences of *Shigella* infection in children to inform the value proposition for an effective vaccine.

**Methods:**

We searched PubMed and Embase for studies published from January 01, 1980 to December 12, 2022 and conducted in low- and middle-income countries that included longitudinal follow-up after *Shigella* detection among children aged <5 years, irrespective of language. We collected data on all outcomes subsequent to *Shigella* detection, except mortality.

**Results:**

Of 2627 papers identified, 52 met inclusion criteria. The median sample size of children aged <5 years was 66 (range 5-2172). Data were collected in 20 countries; 56% (n = 29) of the publications included Bangladesh. The most common outcomes related to diarrhea (n = 20), linear growth (n = 14), and the mean total cost of a *Shigella* episode (n = 4; range: $ 6.22-31.10). Among children with *Shigella* diarrhea, 2.9-61.1% developed persistent diarrhea (≥14 days); the persistence was significantly more likely among children who were malnourished, had bloody stool, or had multidrug-resistant *Shigella*. Cumulative *Shigella* infections over the first 2 years of life contributed to the greatest loss in length-for-age z-score.

**Conclusion:**

We identified evidence that *Shigella* is associated with persistent diarrhea, linear growth faltering, and economic impact to the family.

## Highlights

• Systematic review of longitudinal *Shigella* outcomes in children.
• *Shigella* is associated with continued diarrhea and linear growth faltering.
• There is a need for standardized measurement and reporting of *Shigella* outcomes.

## Introduction

*Shigella* is a highly transmissible enteric pathogen, which causes an estimated 68,000 deaths in children aged <5 years each year [1] and is indirectly responsible for an additional 13,600 deaths from *Shigella*-associated linear growth faltering or stunting [2]. The mortality rates from *Shigella* have declined substantially over the last few decades due to the apparent disappearance of the highly virulent Shiga toxin-producing *Shigella dysenteriae* 1 serotype, measles vaccination, antibiotics, improvements in nutritional status, and economic development [3], [4], [5]. Despite these gains, antibiotic resistance to first and secondline antibiotics that have historically been effective in reducing disease severity, diarrhea duration, and pathogen excretion threatens the progress that has been made in reducing *Shigella* mortality [6].

In addition to its contribution to childhood mortality, *Shigella* is responsible for substantial morbidity among children aged <5 years. This gram-negative bacterium is often the leading cause of moderate-to-severe diarrhea (MSD) and is the leading cause of dysentery among children aged <5 years living in low- and middle-income countries (LMICs) [7,8]. The incidence of *Shigella* acute diarrhea ranges from 1 per 100 child-years to 75.1 per 100 child-years among children in LMICs [7,9,10]. *Shigella* infections, in the presence and absence of diarrhea, also contribute to linear growth faltering [11,12], likely through a mechanism involving environmental enteric dysfunction (EED) [9,13]. EED and linear growth faltering both have links to poor longer-term outcomes, including delayed cognitive development, poor school performance, and reduced economic potential [14], [15], [16]. *Shigella* infections also pose a significant financial burden on families and health systems due to the treatment/hospitalization cost of *Shigella* diarrhea [17,18] and from potential decreased economic/earning potential from the longer-term outcomes of *Shigella* [19].

Based on the clinical severity, disease burden, links to longer-term outcomes, and the emergence of antimicrobial resistance, *Shigella* is a priority for vaccine development in the target population of young children living in LMICs [20]. Vaccines targeting the most common *Shigella flexneri* serotypes and *Shigella sonnei* are in development [21,22]. As pediatric *Shigella* vaccines move toward licensure and policy makers consider vaccine introduction, there is a need to synthesize evidence on the long-term consequences of *Shigella* to aid global and country decision-making to support vaccine adoption [20,23]. We conducted a systematic review of the consequences of *Shigella* infection among children in LMICs to help characterize the potential value of a *Shigella* vaccine.

## Methods

We conducted a systematic review following the Preferred Reporting Items for Systematic Reviews and Meta-Analyses guidelines [24] to identify literature on the consequences of *Shigella* infection in children aged <5 years in LMICs. We aimed to gather data on the breadth of sequelae attributable to *Shigella* infection among young children, including but not limited to diarrhea persistence, linear growth faltering, ponderal growth faltering, neurodevelopmental delay, economic impacts, immune response, and systemic and enteric inflammation. In addition to characterizing the evidence and direction of effect, we sought to identify evidence gaps that could be addressed in future research studies.

### Search strategy and selection criteria

We searched PubMed and Embase for articles published from January 01, 1980 to December 12, 2022 that indicated longitudinal follow-up of children after detection of *Shigella* in fecal samples or blood by any laboratory method. We included terms that described LMICs, as well as the names of all countries categorized as LMICs by the World Bank in 2020 (see Appendix 1 for full search strings).

We included clinical trials and observational studies that followed up at least five children with *Shigella* detected for any duration beyond 1 hour, regardless of symptoms. We restricted to studies conducted in LMICs that reported outcome data for children aged <5 years (0-60 months) to focus on the population with the highest morbidity and mortality burden attributed to *Shigella* [1]. We excluded cross-sectional studies and outcomes that were assessed contemporaneously with *Shigella* detection. Conference abstracts were included if they met other inclusion criteria and contained outcome data. We translated non-English publications using DeepL Translator (Cologne, Germany) or Google Translate.

Two reviewers (FA, MD, or TL) independently screened the title and abstract of each article for eligibility using Covidence (Veritas Health Innovation, Melbourne, Australia). Any disagreements were resolved by a third reviewer (PP) or through group discussion and consensus. If a decision could not be made using the information available in the abstract or if no abstract was available, the article was passed to full-text review. The same methods (dual review and conflict resolution using Covidence) were used during full-text review. The review's International prospective register of systematic reviews registration number is CRD42021241169 (link).

### Data analysis

The summary data were abstracted from full-text reports of included publications. We abstracted information on the original study design and methodology (e.g., length of follow-up, inclusion criteria), the location of study, the number of children and/or stools with *Shigella* detected, laboratory method of detection, *Shigella* species identified, co-infections, and funding source. For each outcome identified, we abstracted the method of measurement, time point of measurement or duration of follow-up, any adjustment variables, and the effect estimate. All longitudinal outcomes were abstracted except mortality because this outcome was recently summarized in a systematic review of case fatality rates for common diarrheal pathogens [25]. Clinical characteristics and outcomes reported only at medical presentation or study enrollment were not abstracted because it was not possible to determine temporality in relation to *Shigella* detection. Data from randomized trials were abstracted for each randomization arm; the measures of excess risk comparing randomization arms were not abstracted unless they compared children with and without *Shigella* detected.

Because all data in this review were treated as a cohort study (*Shigella* as the exposure), we did not feel it would be relevant to assess the risk of bias for the original study design (e.g., randomized control trial) nor would it be possible to uniformly apply a risk of bias assessment tool to the variety of designs included in this review because many questions are not suited to our included outcomes. Instead, we conducted a quality assessment of included studies using a modified version of a composite quality construct based on the Strengthening the Reporting of Observational Studies in Epidemiology statement [26], which was developed and implemented previously [27]. In this assessment, each article was awarded points (10 maximum) for satisfying components of the methods section of the Strengthening the Reporting of Observational Studies in Epidemiology statement checklist, which includes an assessment of efforts to address potential sources of bias (Appendix 2). A rating of ‘poor’ was assigned to articles with zero to four points, ‘fair’ with five to seven points, and ‘good’ with eight to 10 points. As part of our quality assessment, we reviewed information contained within a given publication, as well as the text of referenced articles as needed.

Data abstraction was performed by a single reviewer (FA, MD, or TL) and quality checks were performed on a random subset of the data (20%). The study data were collected and managed using Research Electronic Data Capture tools hosted at the University of Washington Institute of Translational Health Sciences [28,29]. We performed a descriptive summary of the study characteristics and longitudinal outcomes. The definitions of acute and persistent diarrhea were accepted from included studies, but the review adapted the distinction of <14 and ≥14 days, distinguishing the two as described in WHO diarrhea treatment guidelines [30]. We intended to conduct a meta-analysis for any outcomes that were reported consistently by more than two studies. Due to heterogeneity in the measurement methods, comparison groups, and follow-up duration, we report a narrative summary of the evidence for each outcome.

## Results

**Figure 1:**
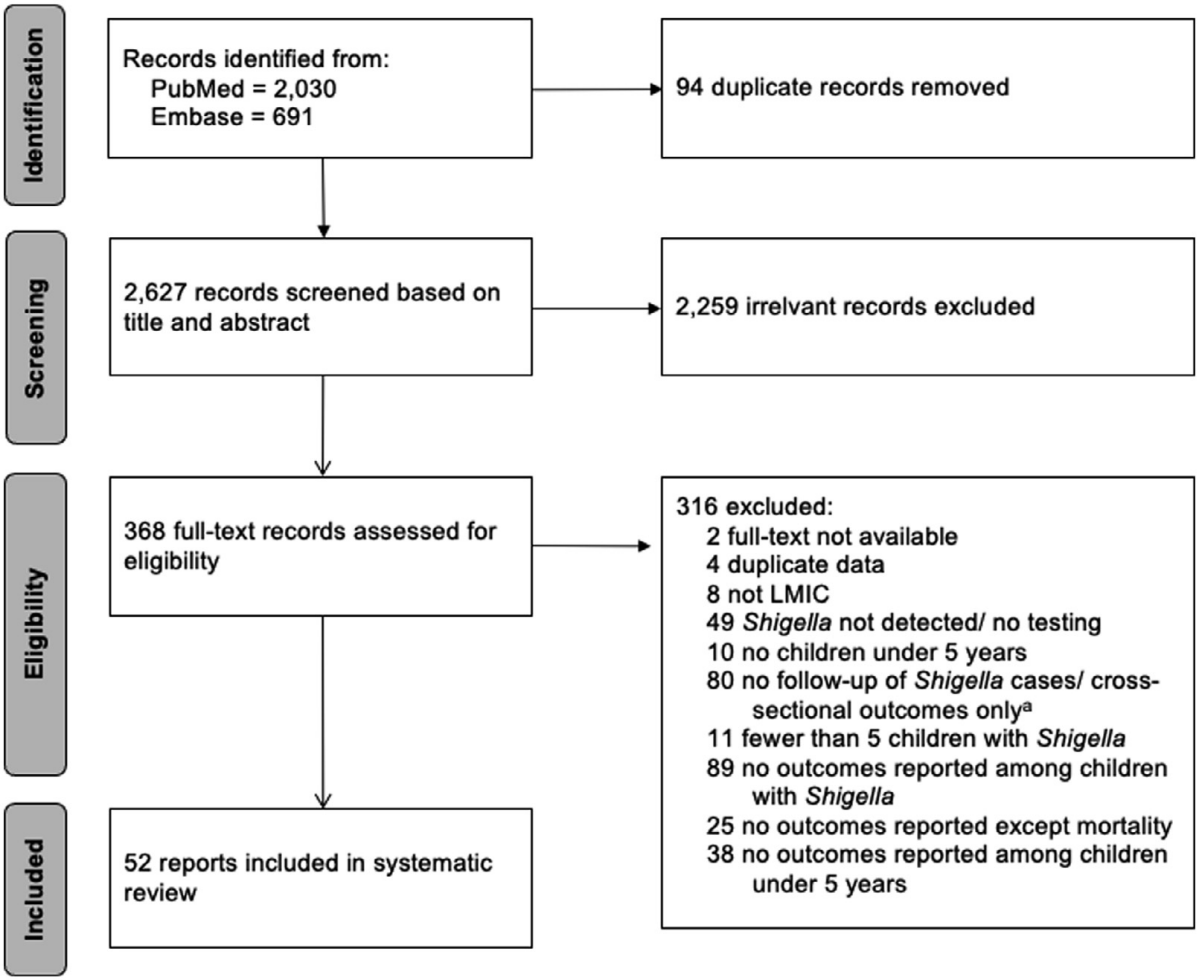
Study selection (preferred reporting items for systematic reviews and meta-analyses [PRISMA] diagram). ^a^Studies that were excluded for “no follow-up of *Shigella* cases/cross-sectional outcomes only” include some studies that were longitudinal in nature, but presented outcomes cross-sectionally such that the likelihood of longitudinal outcomes given *Shigella* infection could not be determined (e.g., given all children with an outcome, the percent of children that had *Shigella* infection) either from direct interpretation of tables or through back calculations. Abbreviations: LMIC, low– or middleincome country.

Our final search identified 2627 potentially eligible records from PubMed and Embase after deduplication (Figure 1). We completed the dual review of titles and abstracts passing 368 (14%) publications to full-text review, of which 52 met the inclusion criteria (Figure 1). The 316 studies excluded at full-text review are described in Appendix 3. The key characteristics of the 52 included articles are shown in Table 1 and summarized in Table 2. The data on *Shigella* outcomes were collected in 20 different countries; although 56% (n = 29) of the publications were from studies conducted at least partially in Bangladesh. There were 13 publications from studies conducted on the African continent. Five publications reported data from multiple countries either collected as part of the Etiology, Risk Factors, and Interactions of Enteric Infections and Malnutrition and the Consequences for Child Health and Development (MAL-ED) cohort study (n = 3) [31] or the Global Enteric Multicenter Study (GEMS; n = 2) [32]. The study designs included cohort studies (n = 19), randomized trials (n = 13), disease surveillance (n = 11), and case-control studies (n = 9) (Table 2).

**Table 1:** Characteristics of included publications (n = 52).

Study	Country	Region	Study type	Dates^b^	Age range^c^ (months)	Population description	Primary *Shigella* detection method	# of children with *Shigella*	Diarrhea/ asymptomatic stools	Outcomes
Abu-Elyazeed *et al.*[40]	Egypt	Eastern Mediterranean	Cohort	1995 - 1998	0-36	Children in cohort without congenital abnormalities or hospitalization history	Culture	101	Diarrhea	Diarrhea, repeat *Shigella* infection(s)
Ahmed *et al.*[39]	Bangladesh	South-East Asia	Cohort	1987 - 1989	0-59	Children who were neighborhood contacts of Shigella cases and had diarrhea between 24 hours and 18 days of follow-up	Culture	104	Diarrhea	Diarrhea
Anders *et al.*[66]	Vietnam	South-East Asia	Cohort	2009 - 2013	0-12	Infants in birth cohort	qPCR	108	Diarrhea	Repeat *Shigella* infection(s)
Andersson *et al.*[62]	Tanzania	Africa	Cohort	Apr 2011 - Jul 2011	2-59	Children with history of loose stools and fever	PCR	42	Diarrhea	Pathogen clearance
Ballard *et al.*[41]	Peru	The Americas	Case-control	Oct 2013 – May 2015	0-59	Children seeking care for acute gastroenteritis and community controls	Culture	23	Diarrhea	Diarrhea
Baqui *et al.*, [38]	Bangladesh	South-East Asia	Surveillance with case follow-up	May 1988 - Apr 1989	0-59	Children in community-based cohort	Culture	Not specified^a^	Diarrhea	Duration of *Shigella* excretion
Black *et al.*[43]	Bangladesh	South-East Asia	Cohort	Mar 1978 - Mar 1979	2-60	Children in community-based cohort	Culture	Not specified^a^	Diarrhea	Diarrhea
Black *et al.*[42]	Bangladesh	South-East Asia	Cohort	Mar 1978 - Mar 1979	2-48	Children in community-based cohort	Culture	Not specified^a^	Diarrhea	Diarrhea
Black *et al.*[49]	Bangladesh	South-East Asia	Cohort	Mar 1978 - Mar 1979	2-48	Children in community-based cohort	Culture	56	Diarrhea	Linear growth, weight gain
Butler *et al.*[80]	Bangladesh	South-East Asia	Surveillance with case follow-up	Jul 1975 - Jun 1980	0-59	Children admitted to hospital with confirmed *Shigella* infection	Culture	2,172	Diarrhea	Leukemoid reaction
Cravioto *et al.*[44]	Mexico	The Americas	Surveillance with case follow-up	Aug 1985 - Feb 1987	0-12	Children in birth cohort	Culture	11	Both	Diarrhea
Das *et al.*[18]	Bangladesh	South-East Asia	Surveillance with case follow-up	Jan 2010 - Dec 2012	0-59	All children with diarrhea in surveillance area at tertiary level hospital	Culture	518	Diarrhea	Economic outcomes
Das *et al.*[57]	Bangladesh	South-East Asia	Case-control	Dec 2007 – Mar 2011	0-59	Children brought to health centers with MSD and community controls (enrolled in GEMS)	Culture	591	Diarrhea	Hospitalization, linear growth, ponderal growth, economic outcomes
Donowitz *et al.*[54]	Bangladesh	South-East Asia	Cohort	Jun 2014 - Mar 2016	0-24	Children in birth cohort	qPCR	Not specified^a^	Diarrhea	Linear growth, neurodevelopmental outcomes
Dutta *et al.*[81]	India	South-East Asia	Surveillance with case follow-up	Not specified	0-59	Children admitted to hospital with acute diarrhea or dysentery for <3 days	Culture	46	Diarrhea	Diarrhea
Dutta *et al.*[36]	India	South-East Asia	Surveillance with case follow-up	Jan 1985 - Dec 1988	6-59	Children admitted to hospital with acute diarrhea or dysentery who did not receive antibiotics prior to hospitalization	Culture	192	Diarrhea	Diarrhea
Echeverria *et al.*[68]	Thailand	South-East Asia	Case-control	Not specified	10-48	Children with confirmed *Shigella* with fever, abdominal cramping, and bloody diarrhea	Culture	19	Diarrhea	Antibody response
Fujita *et al.*[82]	Kenya	Africa	Case-control	Sep 1986 - Aug 1987	12-59	Children visiting health center with acute infectious diarrhea	Culture	5	Diarrhea	Stool pH/water content
Gaensbauer *et al.*[61]	Guatemala	The Americas	RCT	Mar 2015 - Jan 2016	6-35	Children with moderate or severe diarrhea enrolled in an RCT of a nutritional product	PCR	112	Diarrhea	Pathogen clearance
George *et al.*[50]	Bangladesh	South-East Asia	Cohort	2014	6-30	A random subset of children enrolled in GEMS	qPCR	71	Both	Linear growth, ponderal growth/weight gain
Guh *et al.*[58]	China	Western-Pacific	Surveillance with case follow-up	Jan 2002- Dec 2002	0-59	Children with diarrhea or dysentery and confirmed shigellosis presenting for healthcare	Culture	55	Diarrhea	Economic outcomes
Henry *et al.*[35]	Bangladesh	South-East Asia	Surveillance with case follow-up	Mar 1987 - Feb 1989	0-71	Children in community-based cohort	Culture	213	Both	Diarrhea
Househam *et al.*[83]	South Africa	Africa	Cohort	Not specified	1.5-12	Children admitted to rehydration facility without associated parenteral infection	Culture	31	Diarrhea	Diarrhea
Huskins *et al.*[84]	Bangladesh	South-East Asia	Surveillance with case follow-up	Jan 1984 - Dec 1988	0-3	Children hospitalized with confirmed *Shigella* infection	Culture	159	Diarrhea	Hospital discharge status
Huttly *et al.*[37]	Bangladesh	South-East Asia	Surveillance with case follow-up	Mar 1984 - Dec 1987	0-59	Children in community-based environmental intervention trial	Culture	Not specified^a^	Diarrhea	Diarrhea
Kabir *et al.*[56]	Bangladesh	South-East Asia	RCT	Not specified	24-59	Children from outpatient department with *Shigella* detected, treated for 5 days with effective antibiotic	Culture	69	Diarrhea	Linear growth, ponderal growth/weight gain
Kabir *et al.*[48]	Bangladesh	South-East Asia	RCT	Not specified	24-59	Children with bloody mucoid stools for <5 days enrolled in RCT (Kabir *et al.*[56] )	Culture	59	Diarrhea	Diarrhea, linear growth, ponderal growth/weight gain, subsequent illness
Khan *et al.*[65]	Bangladesh	South-East Asia	Surveillance with case follow-up	1973 - 1980	0-59	Children with family member with Shigellosis	Culture	132	Diarrhea	Duration of *Shigella* excretion
Luoma *et al.*[51]	Malawi	Africa	Cohort	Feb 2011 – Aug 2012	18-24	Seemingly healthy children participating in an extension to a nutrient supplement trial	qPCR	Not specified^a^	Asymptomatic	Linear growth
Mazumder *et al.*[69]	Bangladesh	South-East Asia	RCT	Not specified	12-48	Malnourished children hospitalized with blood in stool for <72 hours	Culture	23	Diarrhea	Diarrhea, nutrient absorption
Mazumder *et al.*[45]	Bangladesh	South-East Asia	RCT	Not specified	12-48	Malnourished children with blood in stool for <96 hours	Culture	75	Diarrhea	Ponderal growth/weight gain
Mitra *et al.*[46]	Bangladesh	South-East Asia	Cohort	May 1995 - Dec 1995	5-60	Children hospitalized with blood in stool and with no history of antibiotics or vitamin A supplementation	Culture	66	Diarrhea	Diarrhea, hospitalization, ponderal growth/weight gain, serum retinol concentration
Nasrin *et al.*[12]	Bangladesh, The Gambia, India, Kenya, Mali, Mozambique, Pakistan	South-East Asia, Africa	Case-control	2007-2011	0-59	Children with moderate-to-severe diarrhea enrolled in GEMS	Culture	Not specified^a^	Diarrhea	Linear growth
Ndungo *et al.*[71]	Malawi	Africa	Cohort	Feb – Nov 2016	0-24	Children enrolled in Malaria birth cohort study and sex- and age-matched controls	qPCR	30	Both	Microbiome composition
Perin *et al.*[85]	Bangladesh	South-East Asia	Case-control	2014 - 2015	6-31	Children in cohort	16s sequencing	Not specified^a^	Both	Linear growth, ponderal growth/weight gain
Platts-Mills *et al.*[55]	Tanzania	Africa	Cohort	Dec 2009	1-12	Children in birth cohort with diarrhea	qPCR	19	Diarrhea	Linear growth
Platts-Mills *et al.*[86]	Bangladesh	South-East Asia	Case-control	2009 - 2012	6-23	Children participating in an intervention with WAZ <-2 (cases) and WAZ >-1 (controls)	qPCR	139	Diarrhea	Malnutrition
Platts-Mills *et al.*[8]	Niger	Africa	RCT	Oct 2014 – Dec 2017	0-23	Children who received 3 doses of rotavirus vaccine or placebo without RCT protocol violation	qPCR	147	Diarrhea	Diarrhea
Rahman *et al.*[70]	Bangladesh	South-East Asia	RCT	Not specified	6-35	Children with bloody mucoid stools for <5 days and no history of potentially effective drugs	Culture	66	Diarrhea	Nutritional intake
Cruz *et al.*[67]	Guatemala	The Americas	Cohort	Not specified	0-35	Children in community-based cohort	Culture	126	Both	Diarrhea, repeat *Shigella* infection(s), nutritional intake
Rampengan *et al.*[47]	Indonesia	South-East Asia	Cohort	Jul 1974 - Jun 1976	0-59	Children hospitalized with dysentery and confirmed *Shigella* infection	Culture	46	Diarrhea	Diarrhea, duration of fever, hospitalization
Raqib *et al.*[60]	Bangladesh	South-East Asia	RCT	Not specified	12-59	Moderately malnourished children with acute shigellosis	Culture	56	Diarrhea	Antibody response, EED, inflammation
Riewpaiboon *et al.*[17]	Thailand	South-East Asia	Case-control	May 2002 - Apr 2003	0-59	Children presenting to health center with shigellosis	Culture	130	Diarrhea	Economic outcomes
Rodriguez *et al.*[63]	Mexico	The Americas	RCT	Jan 1987 - Jul 1988	2-59	Children in RCT who visited hospital with bloody diarrhea <5 days and without history of potentially effective drugs	Culture	35	Diarrhea	Pathogen clearance
Rogawski *et al.*[11]	Bangladesh, Brazil, India, Nepal, Pakistan, Peru, South Africa, Tanzania	South-East Asia, Africa, the Americas	Cohort	2009 - 2012	0-60	Children in MAL-ED birth cohort: infants from singleton pregnancies without very low birth weight, congenital disease, or severe neonatal disease	qPCR	Not specified^a^	Both	Linear growth, ponderal growth/weight gain
Rogawski McQuade *et al.*[9]	Bangladesh, Brazil, India, Nepal, Pakistan, Peru, South Africa, Tanzania	South-East Asia, Africa, the Americas	Cohort	2009 - 2012	0-24	Children in MAL-ED birth cohort	Culture	Not specified^a^	Diarrhea	Diarrhea, fever in subsequent *Shigella*-attributable diarrhea episode, hospitalization
Rogawski McQuade *et al.*[52]	Brazil, South Africa, Tanzania	Africa, the Americas	Cohort	2009-2012	0-24	Children in MAL-ED birth cohort	qPCR	Not specified^a^	Asymptomatic	Linear growth, neurodevelopmental outcomes
Roy *et al.*[33]	Bangladesh	South-East Asia	RCT	1999 - 2002	12-59	Moderately malnourished children with shigellosis dysentery	Culture	56	Diarrhea	Diarrhea, linear growth, ponderal growth/weight gain, subsequent illness
Schnee *et al.*[53]	Bangladesh	South-East Asia	RCT	2011 - 2012	0-24	Children in birth cohort with diarrhea	qPCR	Not specified^a^	Diarrhea	Inflammation, linear growth
Taylor *et al.*[34]	Thailand	South-East Asia	RCT	Nov 1984 - Jan 1985	2-60	Children in drug trial with diarrhea and fever, vomiting, or colic for <24 hours	Culture	21	Diarrhea	Diarrhea
Versloot *et al.*[64]	Malawi	Africa	RCT	Jan 2013 - Jul 2013	8-59	Children in an RCT who were hospitalized for complicated severe acute malnutrition	PCR	19	Both	Pathogen clearance
Zimmermann *et al.*[59]	Bangladesh, The Gambia, India, Kenya, Mali, Mozambique, Pakistan	South-East Asia, Africa	Case-control	Dec 2007 - Mar 2011	0-59	Children with acute diarrhea (any severity) enrolled in GEMS	Culture	1,736	Diarrhea	Economic outcomes

Abbreviations: EED, environmental enteric dysfunction; GEMS, the Global Enteric Multicenter Study; MAL-ED, Etiology, Risk Factors, and Interactions of Enteric Infections and Malnutrition and the Consequences for Child Health and Development; RCT, randomized controlled trial; qPCR, quantitative polymerase chain reaction; MSD, moderate-to-severe diarrhea; WAZ, weight-for-age z-score.

a The number of children with *Shigella* detected was not specified in some studies; see Appendix 4 for the # of *Shigella*-positive stools or diarrhea episodes attributable to *Shigella*, which were used to verify inclusion criteria of 5+ children with *Shigella*.

b The months (if available) and years of participant enrollment.

c The age range of enrolled children for whom outcomes were measured/reported.

**Table 2:** Summary of included publications (n = 52).

Publication characteristic	Number of publications	(%)
**Geographic region**^a^		
South-East Asia	36	69%
Africa	13	25%
The Americas	8	15%
Western-Pacific	1	2%
Eastern Mediterranean	1	2%
**Country**^a^		
Bangladesh	29	56%
India	6	12%
Tanzania	5	10%
Pakistan	4	8%
South Africa	4	8%
Malawi	3	6%
Thailand	3	6%
Other	17	33%
**Study type**		
Cohort	19	37%
Randomized controlled trial	13	25%
Surveillance (with case follow-up)	11	21%
Case-control	9	17%
**Primary *Shigella* detection method**		
Culture	37	71%
qPCR	11	21%
PCR	3	6%
16S sequencing	1	2%
**Number of children with *Shigella***		
Mean	192	
Median (range)	66 (5-2,172)	
**Publication date**		
1980 to 1989	10	19%
1990 to 1999	15	29%
2000 to 2009	6	12%
2010 to present	21	40%
**Reported outcomes**^a^		
Diarrhea-related outcomes	20	38%
Linear growth	14	27%
Other anthropometric measures^b^	10	19%
Economic outcomes	5	10%
Pathogen clearance	4	8%
Repeat *Shigella* infections	4	8%
Systemic inflammation	2	4%
Neurodevelopmental outcomes	2	4%
Gut inflammation, environmental enteric dysfunction	1	2%
Other outcomes	16	31%
**Quality score**		
Poor	1	2%
Fair	16	31%
Good	35	67%

Abbreviations: qPCR, quantitative polymerase chain reaction.

a Categories are not mutually exclusive therefore percentages may exceed 100%

b Includes ponderal growth, weight gain, underweight, malnutrition, etc.

Publications included a median of 66 children with *Shigella*, ranging from five to 2172 (Table 2). Of note, some of the included studies did not specify the number of children with *Shigella* but provided other information that made it possible to estimate the number of children with *Shigella* as being five or more (Appendix 4). Although most publications were among children with *Shigella* diarrhea only, nine (17%) publications also included *Shigella* detected in asymptomatic patients (Table 1). The study setting and initial inclusion criteria varied widely, such as malnourishment, current diarrhea, participation in birth and community-based cohorts or randomized controlled trials, admittance to hospitals, and presentation at health care facilities. Culture was the most common primary *Shigella* detection method (71%), followed by quantitative polymerase chain reaction (21%; Tables 1, 2). Most studies were rated ‘good’ quality (n = 35; 67%), followed by ‘fair’ quality (n = 16; 31%) and ‘poor’ quality (n = 1; 2%) (Appendix 5).

The most commonly reported outcomes of *Shigella* were related to diarrhea (n = 20) and linear growth (n = 14). Other anthropometric measures, such as ponderal growth (e.g., change in weight-for-height z-score [WHZ]) or weight gain (e.g., change in weight or weight-for-age z-score [WAZ]), were reported in 10 studies (Table 2). In each of these categories, fewer than three studies reported on the same outcome using a similar comparison group, thus precluding meta-analyses.

### Diarrhea outcomes

There were three general categories of measurement among studies of diarrhea outcomes: duration of diarrhea measured continuously (n = 9); duration of diarrhea measured categorically (<7 days, 7-<14 days, ≥14 days) and presented as corresponding percentages, odds ratios (ORs), and risk ratios (n = 11); and characteristics of subsequent diarrhea episodes (both *Shigella* and unspecified) that occurred after diarrhea-free days (n = 3). The measurement details are summarized in Table 3.

**Table 3:** Diarrhea outcomes, by measurement and follow-up duration.

Measurement	Follow-up duration	Study	# with *Shigella*	Outcome measurement/ comparison groups	Effect measure
Acute diarrhea					
*Proportion with diarrhea on Day X*					
	3 days	Abu-Elyazeed *et al.*[40]	101	Percent of children with *Shigella* that had diarrhea^a^ lasting 3 or more days, by serotype	All: 56%; *S. flexneri*: 53%; *S. sonnei*: 55%; *S. dysenteriae*: 61%; *S. boydii*: 50%; Mixed serogroups (1 case): 100%
	4 days	Househam *et al.*[83]	31	Probability of acute diarrhea being self-limiting (less than 4 days of treatment in rehydration facility needed before discharge home and no past month or following months admissions to a rehydration facility) given *Shigella* present	0.74; i.e., significantly higher (*P* <0.05) than when Shigella is not present
	5 days	Abu-Elyazeed *et al.*[40]	101	Percent of children with *Shigella* that had diarrhea^a^ lasting 5 days	23%
*Relative proportion/odds of diarrhea on Day X (OR)*					
	3 days	Abu-Elyazeed *et al.*[40]	101	Adjusted OR (95% CI) for *Shigella* diarrhea (as opposed to non-*Shigella* diarrhea) among children with illness duration of 3 or more days, adjusting for fever, vomiting, severe dehydration and bloody stool	1.4 (95% CI: 1.0, 2.0)
Prolonged diarrhea					
*Proportion with diarrhea on Day X*					
	7 days	Rogawski *et al.*[9]	Not specified	The percent of *Shigella*-attributable diarrhea episodes where prolonged diarrhea (7+ days) was present, by year of life	Year 1: 24.3%; Year 2: 17.7%
	7 days	Rogawski *et al.*[9]	Not specified	The percent of *Shigella*-attributable diarrhea episodes where prolonged diarrhea was present (7+ days), by co-infection status	*Shigella* only: 19.5%*;* Viral co-etiology: 16.8%; Bacterial co-etiology: 17.9%; Parasitic co-etiology: 29.4%
	7 days	Roy *et al.*[33]	56	Percent of children with *Shigella* dysentery at baseline who had not recovered by day 7 (defined as children who were 'three or fewer formed stools in a day, were afebrile, did not have visible blood or mucous in stools and did not have abdominal pain or tenderness)	Zinc: 11%; No zinc: 25%
	7 days	Platts-Mills *et al.*[8]	147	Prevalence ratio (95% CI) for prolonged diarrhea (≥7 days) comparing children with diarrhea attributable to *Shigella* vs those not attributable to *Shigella*	1.68 (95% CI: 0.99, 2.87)
	7 days	Taylor *et al.*[34]	21	Proportion of children with *Shigella* diarrhea at baseline that still had diarrhea at day 7	Erythromycin group: (3/8) 38%^b^; control group: (1/7) 14%
*Relative proportion/risk of diarrhea on Day X (RR)*					
	7 days	Rogawski *et al.*[9]	Not specified	The site-adjusted risk ratio (95% CI) comparing the percent of *Shigella-*attributed episodes leading to prolonged diarrhea (7+ days) in the first year compared to the second year of life	1.24 (95% CI: 0.88, 1.74)
	7 days	Rogawski *et al.*[9]	Not specified	The site and age-adjusted risk ratios (95% CI) for prolonged diarrhea (7+ days) comparing *Shigella* episodes with co-etiologies to single etiology	Viral co-etiology: 1.15 (95% CI: 0.83, 1.60); Bacterial co-etiology: 1.18 (95% CI: 0.77, 1.80); RR for parasitic co-etiology not estimated due to small numbers
Persistent diarrhea					
*Proportion with diarrhea on Day X*					
	14 days	Rogawski *et al.*[9]	Not specified	The percent of *Shigella*-attributable diarrhea episodes where persistent diarrhea (14+ days) was present, by year of life	Year 1: 5.6%; Year 2: 2.9%
	14 days	Rogawski *et al.*[9]	Not specified	The percent of *Shigella*-attributable diarrhea episodes where persistent diarrhea (14+ days) was present, by co-etiology status (RRs not calculated due to small numbers of episodes)	*Shigella* only: 3.0%*;* Viral co-etiology: 4.1%; Bacterial co-etiology: 4.7%; Parasitic co-etiology: 0%
	14 days	Henry *et al.*[35]	Not specified	Percent of *Shigella* episodes that had a duration of 14+ days	14.9% (14/94)
	14 days	Dutta *et al.*[36]	192	Percent of children who had diarrhea duration of 14+ days, by nutritional status	Well-nourished: 3.2%, Malnourished: 19.2%; p<0.001
	14 days	Dutta *et al.*[81]	46	Percent of *Shigella* diarrhea with duration of 14+ days, by serotype	*S. flexneri*: 44.8%; *S. dysenteriae* 1: 58.8%
	14 days	Huttly *et al.*[37]	Not specified	Percent of *Shigella* episodes with diarrhea >14 days	61.1%
	1 month	Ahmed *et al.*[39]	104	Percent of *Shigella* diarrhea episodes that became persistent (14+ days) overall, and by presence of blood	Overall: 23% (24/104); bloody: 30%; nonbloody: 18.8%; p>0.05
	1 month	Ahmed *et al.*[39]	104	Percent of *Shigella* diarrhea episodes that became persistent (14+ days) by species	*S. flexneri*: 23.6%; *S. dysenteriae* 1: 26.3%; Other: 20.0%; p>0.05
	1 month	Ahmed *et al.*[39]	104	Percent of *Shigella* diarrhea episodes that became persistent (14+ days) among children with and without multiple antibiotic resistance (ampicillin, trimethoprim-sulfamethoxazole, and nalidixic acid)	With multiple antibiotic resistance: 66.7% (4/6); without: 20.4% (20/98); p<0.05
*Relative risk of diarrhea on Day X (RR, OR)*					
	14 days	Rogawski *et al.*[9]	Not specified	The site-adjusted risk ratio (95% CI) comparing the percent of *Shigella*-attributed episodes leading to persistent diarrhea (14+) in the first year compared to the second year of life	1.32 (95% CI: 0.59, 2.93)
	1 month	Ahmed *et al.*[39]	104	Age-adjusted RR (95% CI) of persistent diarrhea (14+ days) comparing *Shigella*-positive to *Shigella*-negative diarrhea episodes, overall and by the presence of blood	Overall: 1.83 (95% CI: 1.19, 2.81; *P* <0.01); Bloody diarrhea: 1.06 (95% CI: 0.60, 1.86; *P* >0.05); Nonbloody diarrhea: 2.31 (95% CI: 1.24, 4.30; *P* <0.01)
	1 month	Ahmed *et al.*[39]	104	Age-adjusted RR (95% CI) for persistent diarrhea (14+ days) comparing children who have shigellosis with bloody diarrhea to children who have shigellosis with nonbloody diarrhea	1.64 (95% CI: 0.82, 3.26)
	1 month	Ahmed *et al.*[39]	104	Age-adjusted RR (95% CI) of persistent diarrhea (14+ days) with *S. dysenteriae 1* and other *Shigella* serotypes, compared to risk of persistent diarrhea with *S. flexneri*	RR_dys 1 vs flex_: 1.25 (95% CI: 0.49, 3.18); RR_other serotypes vs flex_: 0.78 (95% CI: 0.34, 1.77)
	1 month	Ahmed *et al.*[39]	104	Age-adjusted RR (95% CI) for persistent diarrhea (14+ days) comparing children with shigellosis with multiple antibiotic resistance (resistant to ampicillin, trimethoprim-sulfamethoxazole, and nalidixic acid) to children with shigellosis without multiple antibiotic resistance	3.76 (95% CI: 1.51, 9.36)
Mean/median duration of diarrhea					
	72 hours	Mazumder *et al.*[69]	23	Mean (SE) number of hours of *Shigella* dysentery in the intervention diet (higher protein and energy) and control diet groups	Control diet: 58 (7.9) hours; Test diet: 62 (9.8) hours
	Until 48 hrs symptom-free	Ballard *et al.*[41]	23	Mean (SD) duration among those with diarrhea	6.8 (1.2) days
	20 days	Black *et al.*[43]	117	Median, mean (SE), and range of duration in days of *Shigella* diarrhea episodes	Median: 7; Mean: 10.7 (1); Range: 1–20+ days
	60 days	Black *et al.*[42]	Not specified	Mean duration (days) of *Shigella* diarrhea in highest and lowest weight-for-length Z score groups	Highest: 6.5 days; Lowest: 21.3 days
	60 days	Black *et al.*[42]	Not specified	Mean (SE) duration in days of *Shigella* diarrhea by anthropometric group	Normal: 12.0 (3.1); Stunted: 13.8 (2.9); Stunted and wasted: 15.4 (4)
	60 days	Black *et al.*[42]	Not specified	Mean (SE) duration in days of *Shigella* diarrhea among children <24 months by relative nutritional status	Weight-for-length ≥90%: 8.8 (2.3); 80-89%: 14.9 (3.1); ≤79%: 22.2 (5). Weight-for-age ≥75%: 11.5 (2.4); 60-74%: 16.1 (2.9); <60%: 15.1 (5.5). Length-for-age 90-94%: 13.9 (3); 85-89%: 16.8 (3.5); <85%: 11.2 (3.4); differences were not statistically significantly different
	6 months	Roy *et al.*[33]	56	Mean duration (days) of diarrhea episodes that occurred in the 6-month follow-up (95% CI) in the zinc group and the control group (no zinc supplementation)	Zinc: 9.8 (95% CI: 6.0, 15.9); No zinc: 7.1 (95% CI: 3.2, 12.6); *P* = 0.1
	12 months	Cravioto *et al.*[44]	11	Mean (SD) duration (days) of moderate-to-severe dysentery among children with *Shigella*	5 (1) days
	Not specified	Roy *et al.*[33]	56	Median days to recovery (range) in the zinc group and the control group (no zinc supplementation)	Zinc: 2 (1–8); No zinc: 4 (1–8); *P* = 0.03
	Not specified	Roy *et al.*[33]	56	Median days to disappearance from blood from stool (range) in the zinc group and the control group (no zinc supplementation)	Zinc: 2 (1–4); No zinc: 4 (2–5); *P* = 0.04
	Not specified	Roy *et al.*[33]	56	Median days to disappearance from mucous from stool (range) in the zinc group and the control group (no zinc supplementation)	Zinc: 2 (1, 4); No zinc: 4 (1, 7); *P* = 0.04
	Not specified	Roy *et al.*[33]	56	Median days to resolution of straining (range) in the zinc group and the control group (no zinc supplementation)	Zinc: 2 (1, 6); No zinc: 2 (1, 5); *P* = 0.5
	Not specified	Mitra *et al.*[46]	66	Mean days (SD) until no visible blood in stool (days)	*S. dysenteriae*: 2.9 (1.8); Other *Shigella*: 0.8 (0.7)
	Not specified	Rampengan *et al.*[47]	46	Mean duration (days) of diarrhea during hospitalization	5.8 days
	Not specified	Abu-Elyazeed *et al.*[40]	101	Mean duration (days) of illness^a^	4 days
Subsequent diarrhea					
	6 months	Roy *et al.*[33]	56	Mean number of diarrhea episodes during the 6-month follow-up (95% CI) following an episode of *Shigella* diarrhea comparing children randomized to zinc group vs control group (no zinc supplementation)	Zinc: 2.2 (95% CI: 1.6, 4.1); No zinc: 3.3 (95% CI: 2.7, 4.1); *P* = 0.03
	6 months	Kabir *et al.*[48]	59	Number of diarrhea episodes per child in the 6-month follow-up period among children who received 14 days of high-protein diet and those who received standard-protein diet and the RR (95% CI) comparing standard to high protein.	High protein: 1.9 episodes/child; Standard-protein: 2.3 episodes/child; RR : 1.19 (95% CI: 0.76, 1.85)
	2 years	Rogawski *et al.*[9]	Not specified	Among children who had more than one *Shigella*-attributable diarrhea episode, the percent of subsequent episodes that were severe (CODA score 4+) and the site and age-adjusted risk ratio for severe diarrhea comparing the first episode to subsequent episodes (95% CI)	25.8%; RR: 1.08 (95% CI: 0.82, 1.41)
	2 years	Rogawski *et al.*[9]	Not specified	Among children who had more than one *Shigella*-attributable diarrhea episode, the percent of subsequent episodes with blood and the site and age-adjusted risk ratio for bloody diarrhea comparing the first episode to subsequent episodes (95% CI)	14.9%; RR: 0.81 (95% CI: 0.55, 1.20)
	2 years	Rogawski *et al.*[9]	Not specified	Among children who had more than one *Shigella*-attributable diarrhea episode, the percent of subsequent episodes that were prolonged (7+ days) and the site and age-adjusted risk ratio for prolonged diarrhea comparing the first episode to subsequent episodes (95% CI)	13.7%; RR: 1.13 (95% CI: 0.78, 1.64)
	2 years	Rogawski *et al.*[9]	Not specified	Among children who had more than one *Shigella*-attributable diarrhea episode, the percent of subsequent episodes that were persistent (14+ days) and the site and age-adjusted risk ratio for persistent diarrhea comparing the first episode to subsequent episodes (95% CI)	1.6%; RR: 1.75 (95% CI: 0.67, 4.59)
	2 years	Rogawski *et al.*[9]	Not specified	Among children who had more than 1 *Shigella*-attributable diarrhea episode, the percent of subsequent episodes with high frequency (>6 loose stools in 24 hours) and the site and age-adjusted risk ratio comparing the first episode to subsequent episodes (95% CI)	19%; RR: 1.21 (95% CI: 0.89, 1.63)

Abbreviations: CI, confidence interval; OR, odds ratio; RR, relative risk; SE, standard error; SEM, Standard error of the mean; CODA, a diarrheal severity score (Community Diarrhea).

a "Illness" was presumed to mean diarrhea because stool samples were taken when diarrheal episodes were detected.

b Based on results in Taylor *et al.*[34] Table 3 (there is a discrepancy in number of children with *Shigella* spp. isolated on day 0 in the erythromycin group reported in results text and in Table 3).

Briefly, based on three studies, between 11% and 25% of children with *Shigella* diarrhea went on to develop prolonged diarrhea (duration 7-<14 days) [9,33,34], with no statistically significant difference in risk by age (1 year vs 2 years) or co-infection status [9]. Six studies reported on persistent diarrhea (duration ≥14 days) and in these studies, 2.9-61.1% of children with *Shigella* diarrhea developed persistent diarrhea [9,[35], [36], [37], [38], [39]]. Two of these studies reported on risk factors of diarrhea persistence among *Shigella* diarrhea cases, with a statistically significantly higher likelihood of persistence among children who were malnourished (malnourished: 19.2% vs well-nourished: 3.2%) [36], had blood in stool (bloody: 30% vs nonbloody: 19%) [39], or had multidrug-resistant *Shigella* (multidrug resistant: 66% vs not multidrug resistant: 20%) [39]. Of note, a study comparing likelihood of persistent diarrhea between children with *Shigella-*positive diarrhea compared with *Shigella*-negative diarrhea found *Shigella* to be significantly associated with persistent diarrhea (relative risk: 1.83; 95% confidence interval [CI]: 1.91, 2.81) [39]. Similarly, another study reported a longer duration of diarrhea in children with *Shigella* diarrhea than those with other causes of diarrhea (OR of duration longer than 3 days: 1.4; 95% CI: 1.0-2.0) [40]. Across the studies, the continuously measured mean duration of diarrhea ranged from 2 to 22.2 days, with substantial variation by intervention status in trials and anthropometric groups [33,[40], [41], [42], [43], [44], [45], [46], [47]]. There was wide heterogeneity in the information presented on subsequent new diarrhea episodes (Table 3) [9,33,48].

### Growth outcomes

**Figure 2:**
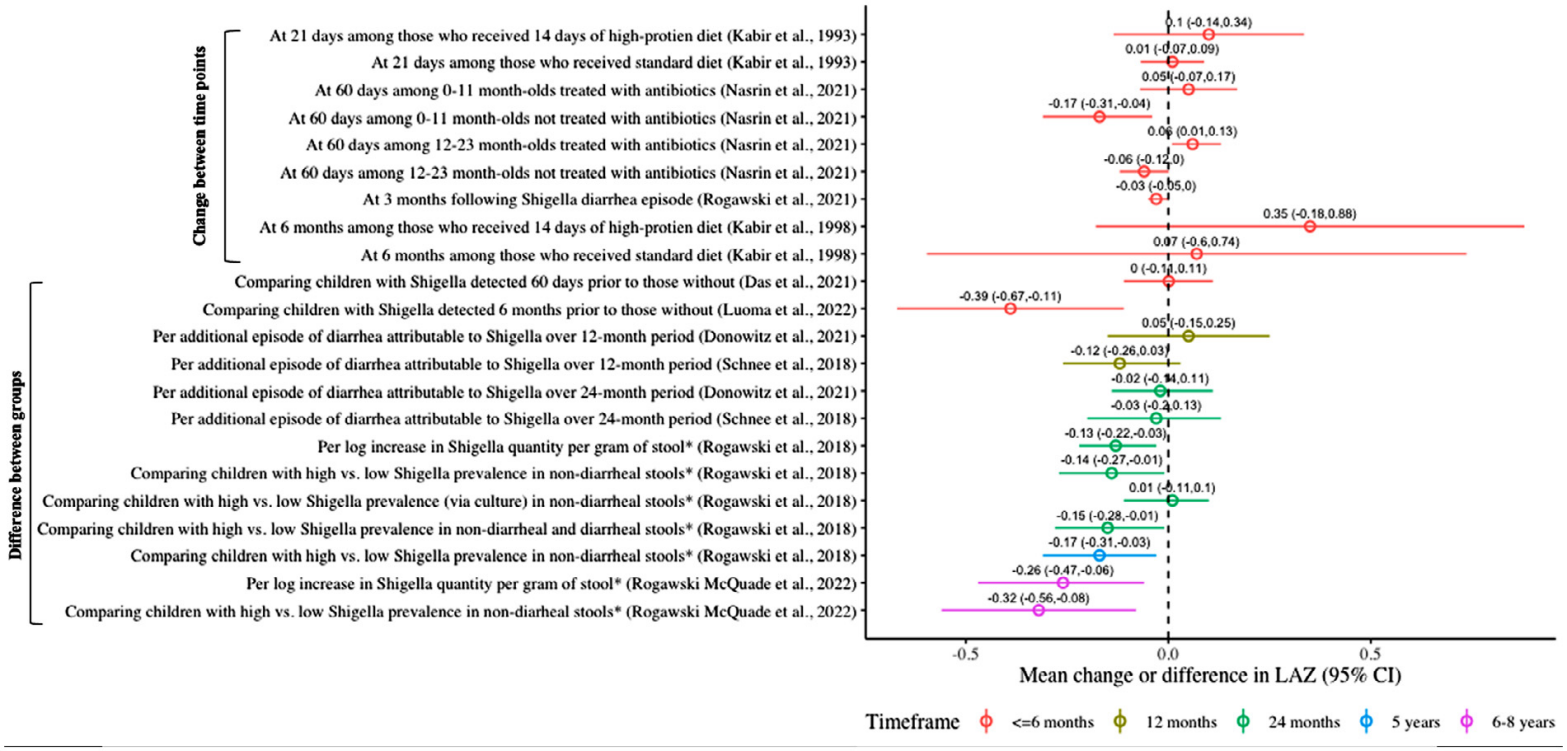
Mean change or difference in LAZ by comparison group and duration of follow-up. *Shigella prevalence or quantity was assessed over a 24-month period. “High” was defined as 90^th^ percentile and “low” as 10^th^ percentile. Abbreviations: CI, confidence interval; LAZ, length-for-age z-score.

Six of 14 studies meeting the inclusion criteria found a statistically significant decrease in linear growth associated with *Shigella* in diarrheal [11,12,49,50] and nondiarrheal [11,51,52] stools (Table 4). There was substantial heterogeneity in measurement time points (ranging from 21 days to 8 years) and comparison groups (Table 4). Linear growth was commonly operationalized as the mean change in the length-for-age z-score (LAZ) between two time points (n = 3) or the difference in LAZ between two groups, defined by presence/absence of *Shigella* or high/low quantity of *Shigella* (n = 7). The effect estimates from these studies are summarized in Figure 2. The differences in LAZ comparing high with low *Shigella* prevalence in nondiarrheal stools ranged from -0.14 (95% CI: -0.27, -0.01) at 2 years to -0.32 (95% CI: -0.56, -0.08) at 6-8 years [11,52]; the mean differences in LAZ per attributable episode of *Shigella* diarrhea ranged from -0.12 (95% CI: -0.26, 0.03) [53] to 0.05 (95% CI: -0.15, 0.25) [54]. Two studies reported on the impact of *Shigella* diarrhea on linear growth at 3 months after diarrhea: one study found a statistically significant average loss of -0.03 (95% CI: -0.05, -0.00) in LAZ [11], whereas another study found no difference in the 3-month LAZ associated with *Shigella* quantity during the diarrheal episodes [55]. In GEMS, *Shigella* episodes not treated with antibiotics led to greater declines in linear growth than treated episodes among children aged <24 months [12]. Another study found that Malawian children with *Shigella* detected at age 18 months had, on average, 0.39 lower LAZ at 24 months than children without *Shigella* detected [51]. George *et al.* [18] found *Shigella* infection to be associated with a two-fold increase in the odds of stunting (defined as height-for-age z-score <-2) at 9 months of follow-up (OR: 2.01; 95% CI: 1.02, 3.93) [50], and Black *et al.* [7,8] reported a statistically significant association between the periods of *Shigella* diarrhea and change in height-for-age compared with a village standard between the beginning and end of the study period [49].

**Table 4:** Linear growth outcomes, by measurement and follow-up time frame.

Outcome	Follow-up duration	Study	# with *Shigella*	Comparison groups	Effect measure
**Mean change in LAZ between two time points**					**Mean △ in LAZ (95% CI)**
	21 days	Kabir *et al.*[56]	69	At 21 days compared to day 1 among those who received 14 days of high-protein diet	+0.1 (SD: 0.12)
	21 days	Kabir *et al.*[56]	69	At 21 days compared to day 1 among those who received standard diet	+0.01 (SD: 0.04)
	∼60 days (49-91)	Nasrin *et al.*[12]	92	At ∼60 days, among children 0-11 months, treated with antibiotic, adjusting for other pathogens	0.05 (−0.07, 0.17)
	∼60 days (49-91)	Nasrin *et al.*[12]	72	At ∼60 days, among children 0-11 months, not treated with antibiotic, adjusting for other pathogens	−0.17 (−0.31, −0.04)
	∼60 days (49-91)	Nasrin *et al.*[12]	282	At ∼60 days, among children 12-23 months, treated with antibiotic, adjusting for other pathogens	0.06 (0.009, 0.13)
	∼60 days (49-91)	Nasrin *et al.*[12]	159	At ∼60 days, among children 12-23 months, not treated with antibiotic, adjusting for other pathogens	-0.06 (-0.12, 0.001)
	∼60 days (49-91)	Nasrin *et al.*[12]	396	At ∼60 days, among children 24-59 months	Non-significant
	3 months	Rogawski *et al.*[11]	NS; 1,469^a^	At 3 months following *Shigella* diarrhea episode	-0.03 (-0.05, -0.00)
	6 months	Kabir *et al.*[48]	59	At 6 months compared to day 1 among those who received 14 days of high-protein diet	+0.35 (SD: 0.27)
	6 months	Kabir *et al.*[48]	59	At 6 months compared to day 1 among those who received standard diet	+0.07 (SD: 0.34)
**Mean difference in LAZ**					
	∼60 days (50-90)	Das *et al.*[57]	591	Comparing children with *Shigella* detected 60 days prior to those without, unadjusted	-0.11 (-0.21, -0.02)
	∼60 days (50-90)	Das *et al.*[57]	591	Comparing children with *Shigella* detected 60 days prior to those without *Shigella* detected, adjusted for confounders, co-infections	0.001 (-0.11, 0.11)
	3 months	Platts-Mills *et al.*[55]	19	At 3 months post-diarrhea comparing high and low quantity of *Shigella* in diarrhea stools	“No specific pathogen quantity in diarrheal stools was significantly associated with poor growth”
	6 months	Luoma *et al.*[51]	NS; 604^a^	At 24 months comparing children with *Shigella* detected at 18 months to those without *Shigella* detected	-0.39 (-0.67, -0.11)
	12 months	Donowitz *et al.*[54]	NS; 250^a^	Per additional episode of diarrhea attributable to *Shigella*	+0.05 (-0.15, 0.25)
	12 months	Schnee *et al.*[53]	NS; 125^a^	Per additional episode of diarrhea attributable to *Shigella*	-0.12 (-0.26, 0.03)
	24 months	Donowitz *et al.*[54]	NS; 250^a^	Per additional episode of diarrhea attributable to *Shigella*	-0.02 (-0.14, 0.11)
	24 months	Schnee *et al.*[53]	NS; 125^a^	Per additional episode of diarrhea attributable to *Shigella*	-0.03 (-0.20, 0.13)
	24 months	Rogawski *et al.*[11]	NS; 1,469^a^	Comparing children with high (90^th^ percentile) vs low (10^th^ percentile) *Shigella* prevalence in nondiarrheal stools over 24-month period	-0.14 (-0.27, -0.01)
	24 months	Rogawski *et al.*[11]	NS; 1,469^a^	Comparing children with high (90^th^ percentile) vs low (10^th^ percentile) *Shigella* prevalence in nondiarrheal stools (using culture instead of qPCR)	+0.01 (-0.11, 0.10)
	24 months	Rogawski *et al.*[11]	NS; 1,469^a^	Comparing children with high (90^th^ percentile) vs low (10^th^ percentile) *Shigella* prevalence in nondiarrheal and diarrheal stools over 24-month period	-0.15 (-0.28, -0.01)
	24 months	Rogawski *et al.*[11]	NS; 1,469^a^	Per one log increase in *Shigella* quantity (copy number) per gram of stool over 24-month period	-0.13 (-0.22, -0.03)
	5 years	Rogawski *et al.*[11]	NS; 1,202^a^	Comparing children with high (90^th^ percentile) vs low (10^th^ percentile) *Shigella* prevalence in nondiarrheal stools over 24-month period	-0.17 (-0.31, -0.03)
	6-8 years	Rogawski *et al.*[52]	NS; 451^a^	Per one log increase in Shigella quantity per gram of stool over 24-month period	-0.26 (-0.47, -0.06)
	6-8 years	Rogawski *et al.*[52]	NS; 451^a^	Comparing children with high (90^th^ percentile) vs low (10^th^ percentile) Shigella prevalence in nondiarrheal stools over 24-month period	-0.32 (-0.56, -0.08)
**Risk of Stunting (HAZ <-2)**					
	9 months	George *et al.*[50]	71	Comparing likelihood of stunting during follow-up among those with *Shigella* at baseline to those without, after adjusting for age, age, caregiver educational level, breastfeeding, and family size	OR: 2.01 (1.02, 3.93)
**HAZ**					
	6 months	Kabir *et al.*[48]	59	Mean HAZ at 6 months among those who received a high-protein diet	1.28 (SD: 1.15)
	6 months	Kabir *et al.*[48]	59	Mean HAZ at 6 months among those who received a standard diet	-1.96 (SD: 1.43)
**Other linear growth measures**					
	60 days	Roy *et al.*[33]	56	Mean linear growth per month (cm) among children with *Shigella* in zinc group	0.58 cm
	60 days	Roy *et al.*[33]	56	Mean linear growth per month (cm) among children with *Shigella* in control group	0.65 cm
	1 year	Black *et al.*[49]	56	Regression coefficient for *Shigella* on change in length (cm) or change in length status expressed as change in percentage of the village reference for age from the beginning to the end of the study period	Shigella coefficient had borderline significance (*P* = 0.07), but exact coefficient not reported
	1 year	Black *et al.*, [49]	56	Regression coefficient for *Shigella* on change in length status expressed as change in percentage of the village reference height-for-age from the beginning to the end of the study period	-0.083 (p<0.05)
	1 year	Black *et al.*[49]	56	Regression coefficient for *Shigella* on change in length (cm) (adjusting for age and initial length)	-0.075 cm (p<0.05)
	1 year	Black *et al.*[49]	56	Comparison of the percentage of expected linear growth rates (based on all village children) observed during periods of *Shigella* diarrhea compared to no diarrhea	“Periods with *Shigella* diarrhea had significantly lower growth rates” (*P* <0.01)
	18 months	Perin *et al.*[85]	NS; 68^a^	Comparing children in the lowest tertile of change in HAZ to those in the highest tertile of change in HAZ	Average proportional abundance of *Escherichia/ Shigella*: 0.026 vs 0.030

Abbreviations: CI, confidence interval; LAZ, length-for-age z-score; HAZ, height-for-age z-score; NS, not specified; OR, odds ratio; SD, standard deviation; SE, standard error

a Represents the number of children enrolled in the study because the number with *Shigella* was not specified (results reported as *Escherichia/ Shigella)*.

Additional anthropometric outcomes are summarized in Appendix 6. Seven studies assessed the ponderal growth and weight-for-age, four of which did not have a comparison group without *Shigella* infection nor with low levels of *Shigella* [45,46,48,56]. The MAL-ED study found no significant difference in mean WHZ or WAZ between children with high (90th percentile) and low (10^th^ percentile) *Shigella* prevalence in nondiarrheal stools [11]. Two studies reported on children enrolled in the Bangladesh site of GEMS: one found children with *Shigella* infection had significantly lower WHZ (-0.11; 95% CI: -0.21, -0.001) than children who were *Shigella*-negative after 60 days of follow-up [57], whereas the other found no significant difference in the odds of wasting (WHZ <-2) or underweight (WAZ <-2) at the 9-month follow-up [50] (Appendix 6).

### Cost of diarrhea episode

Five publications estimated the cost of a *Shigella* diarrhea episode (Table 5) [17,18,[57], [58], [59]]. In one of these studies, across seven sites, the mean total household out-of-pocket cost (including inpatient and outpatient medical costs, transportation, and prescriptions) was $10.61 (converted from local currency to 2012 US dollars), ranging from $4.92 in Mozambique to $17.18 in Mali [59]. This same study found no statistically significant difference in the cost between *Shigella* diarrhea and other pathogens. A study from China, which additionally included self-reported out-of-pocket expenses for overnight stays, estimated the mean cost to be $22 for children aged 0-1 year and $31 for 2-5 years, which represented 12% and 18% of the average monthly income, respectively [58]. One study from Bangladesh found *Shigella* episodes to cost an average of 5.7% (range <1-78%) of the household monthly income [18]. Although there was heterogeneity in measurement and adjustment factors across studies, a large proportion of costs were associated with hospitalization or inpatient care.

**Table 5:** Economic outcomes.

Outcome	Study	# with *Shigella*	Outcome measurement	Country	Effect measure(s)	
Cost of *Shigella* episode					Mean (SD)	Median
	Zimmerman *et al.*[59]	1736	Unadjusted, total household out-of-pocket costs (estimated by caregiver) including inpatient and outpatient medical costs, transportation, prescriptions (local currency converted to 2012 USD)	Seven combined	$10.61 (25.64)	$25.64
			Same as above	Gambia	$7.95 (21.77)	$3.54
			Same as above	Mali	$17.18 (18.06)	$12.51
			Same as above	Mozambique	$4.92 (5.26)	$2.96
			Same as above	Kenya	$15.52 (8.71)	$13.87
			Same as above	India	$8.55 (8.51)	$5.27
			Same as above	Bangladesh	$11.17 (11.51)	$6.83
			Same as above	Pakistan	$8.68 (59.63)	$1.62
	Zimmerman *et al.*[59]	1736	Total household out-of-pocket costs (estimated by caregiver) *after adjustment* for co-pathogens, age group, and gender (local currency converted to 2012 USD)	Seven combined	$12.73 (95% CI 11.09, 14.37)	
	Riewpaiboon *et al.*[17]	130	Public treatment cost defined as cost of the visit, hospitalization, dispensing, drug, medical devices, and laboratory (2006 USD)	Thailand	$6.22 (95% CI 0.26, 12.19)	$3.20
	Guh *et al.*[58]	55	Cost of illness by age group including self-reported out-of-pocket expenditures related to treatment and recovery, lab tests, medicines, treatment, and overnight stays (2002 PPP-adjusted USD)	China	Age 0-1 years: $22.00 (35.00) Age 2-5 years: $31.10 (71.10)	
	Das *et al.*[57]	590	Total household cost including direct and indirect medical costs (converted to current USD)	Bangladesh	$4.17 (3.64)	
	Das *et al.*[57]	590	Total household cost including direct and indirect medical costs *by duration of hospital stay* (converted to current USD)	Bangladesh	1-3 days: $5.30; 4+ days: $8.95; p<0.001	
	Das *et al.*[57]	590	Total household cost including direct and indirect medical costs *by age group* (converted to current USD)	Bangladesh	0-11 months: $4.01; 12-23 months: $3.84; 24-50 months: $4.55; *P* = 0.080	
Cost of *Shigella* episode as percent of monthly household income					Mean (SD)	Median (range)
	Das *et al.*[18]	518	Total costs including drugs, consultations, and transportation before and after attending hospital *measured as percent expenditure of monthly household income*	Bangladesh	5.74% (8.55)	3.17% (0.06%-77.8%)
	Guh *et al.*[58]	55	Cost of illness by age group including lab tests, medicines, treatment, and overnight stays, *as percent of average monthly household income* (2002 PPP-adjusted income = $184/month)	China	Age 0-1 years: 12.0% Age 2-5 years: 16.9%	

Abbreviations: CI, confidence interval; SD, standard deviation; USD, U.S. dollars; PPP, purchasing power parity.

### Enteric and systemic inflammation

Three studies reported on the longitudinal markers of gut and/or systemic inflammatory response among children with *Shigella* (Appendix 7). In a study of children with *Shigella* treated with antibiotic therapy and randomly assigned to 14 days of zinc supplementation or control, there were no significant differences in concentrations of innate mediators (myeloperoxidase, superoxidase, nitrate) and cytokines (interleukin-2, interferon-γ) in stool or released from mitogen-stimulated mononuclear cells within or between treatment groups over 30 days of follow-up [60]. Stool interleukin-1ß concentrations and serum C-reactive protein levels significantly decreased at days seven and 30 in both groups [60]. Over 2 years of follow-up, Schnee *et al.* [45] found diarrhea attributable to *Shigella* to be associated with elevated C-reactive protein levels (increase of 0.24 [95% CI: 0.03, 0.49] per diarrhea episode).

### Other outcomes

Two studies assessed neurodevelopmental outcomes but did not find statistically significant associations between the diarrhea episodes attributable to *Shigella* and neurodevelopmental scores for motor, language, or cognitive skills [54] or between *Shigella* prevalence in nondiarrheal stools and reasoning skills, phonemic fluency, or semantic fluency at age 6-8 years [52] (Appendix 7). Four studies assessed the proportion of children with *Shigella* who were no longer shedding pathogen at various time points (6, 14, or 31 days, or at clinical stabilization) overall [2,61], and/or stratified by antibiotic treatment [62,63] and/or nutritional status [62,64]. One study estimated the mean duration of *Shigella* excretion (4.1 days; range 1-12) [65] (Appendix 7). Four studies assessed the proportion of children who had repeat *Shigella* infections (ranging from 8% to 35%) [40,44,66,67]. Additional outcomes, including antigen-specific antibody response [60,68], duration of hospitalization [46,47], subsequent respiratory and febrile illnesses [33,48], nutritional intake [67,69,70], microbiome composition [71], and serum retinol [46], are summarized in Appendix 7.

## Discussion

The World Health Organization recently articulated the need for evidence synthesis of long-term morbidities associated with key enteric pathogens, such as *Shigella* [23]. In this systematic review, we document the consequences of *Shigella* infection and disease in children aged <5 years living in LMICs. We found evidence that *Shigella* was associated with linear growth faltering and persistent diarrhea [9,11,12,16,39,51,52]. There was a substantial economic impact on families with children suffering from *Shigella* diarrhea [17,18,[57], [58], [59]]. Heterogeneity in measurement and presentation of outcomes and differences in comparison groups between studies prohibited quantitative synthesis of the data, highlighting the need for standardizing methods for characterizing and reporting on enteric pathogen sequelae.

*Shigella* is a well-known cause of diarrhea, with moderate and severe forms of diarrhea constituting a substantial financial burden on health care systems and families. Our systematic review added to this evidence base by highlighting the consequences of *Shigella* diarrhea. Notably, children with *Shigella* diarrhea had an average duration of illness of 2-22 days, with wide variation [33,40,41,43,44,46,47,49,69], and children with acute *Shigella* diarrhea were more likely to develop persistent diarrhea than children with acute diarrhea caused by other pathogens [39]. Longer diarrhea duration is associated with poorer health outcomes, including mortality, stunting, and wasting [72,73], and poses a greater burden on health care systems due to its increased need for facility-based care. In the few studies that included the economic consequences of *Shigella,* all were focused on the cost of *Shigella* diarrhea borne by families, which ranged from 1% to 78% of the monthly household income [18,58]. With *Shigella* infections likely having impact on a child's health, even in the absence of diarrhea, assigning an economic value to a *Shigella* vaccine will require additional data estimating the financial impact of *Shigella* sequelae beyond diarrhea.

We found *Shigella* to have modest and inconsistent effects on linear growth. Children who fall off their linear growth trajectories are at substantial risk for stunting, a precursor to poorer school performance, cognitive development, and reduced earning potential [74], [75], [76]. The greatest differences in LAZ were observed in the MAL-ED cohort study evaluating cumulative asymptomatic *Shigella* infections occurring over the first 24 months of life and their impact at 2, 5, and 6-8 years of life, with magnitudes ranging from -0.32 to -0.14 [11,52]. These magnitudes are expected to be high because they are comparing the extremes of *Shigella* infection burden: a high burden of attributable diarrhea episodes (90^th^ percentile) with a low burden (10th percentile). To the best of our knowledge, there is no established threshold for what loss in LAZ translates to an increased risk of stunting or even more deleterious outcomes, such as impaired cognitive development and poor school performance. If infection rather than disease is primarily responsible for growth faltering from *Shigella,* then *Shigella* vaccines will need to induce sterilizing immunity to expect a growth benefit from the vaccine—a tall order for any vaccine. More realistically, and similar to the rotavirus vaccines, a *Shigella* vaccine will prevent more severe presentations of *Shigella* diarrhea [77]; we found *Shigella* diarrhea to have modest and inconsistently statistically significant effects on linear growth, which may be due to confounding by antibiotic use. Notably, the GEMS study found a statistically significant mean loss in LAZ of 0.06-0.17 after an untreated *Shigella* MSD episode in infants and toddlers, respectively [12], magnitudes of association consistent with studies of asymptomatic *Shigella* included in this review [11,52]. *Shigella* vaccine trials including linear growth as a secondary outcome, as has been suggested by recent study design consensus statements [20,78], will be best suited to estimate a causal association between *Shigella* and linear growth deficits.

One of the key pathways by which *Shigella* and other enteric pathogens are hypothesized to impact linear growth is through EED. EED is a syndrome characterized by inflammation and impaired function of the small intestine and has been associated with stunting among children [13]. The biomarkers of EED, such as myeloperoxidase, may be an intermediate marker of *Shigella*’s impact on linear growth, and therefore may be important targets for vaccine probe studies to estimate more quickly the impact of *Shigella* on growth. Although we only identified one study that looked at the biomarkers for EED longitudinally [60], we note that the indicators for EED may be measured cross-sectionally at the time of acute infection and such outcomes would not have met inclusion criteria. Therefore, our review of longitudinal consequences is not well suited to examine the cross-sectional associations between *Shigella* and EED.

This review was subject to several limitations in addition to those already discussed. To inform the value proposition for soon to be available *Shigella* vaccines, it was valuable to limit this review to outcomes reported among children aged <5 years with confirmed *Shigella* detection. However, excluding studies that did not disaggregate children with clinically compatible illness but without *Shigella* confirmation may have disproportionally excluded studies from certain time periods or settings with limited diagnostic capacity. For example, publications from the 1980s summarizing dysentery epidemics suspected to be caused by shigellosis rarely reported outcomes among the subgroup of children with culture-confirmed *Shigella*. In addition, some potentially relevant growth data by Lee and colleagues [79] were excluded because all reported results were aggregated with children aged >60 months. However, this study found a similar magnitude of change in linear growth (-0.081 cm) per *Shigella* diarrhea episode as another study included in this review, Black *et al.* [49], and thus, inclusion would not have changed our conclusions. Although the unpublished data were beyond the scope of this review, an individual-level reanalysis of included studies could provide valuable information on linear growth faltering associated with shigellosis. Finally, this review focused exclusively on LMICs, based on children living in these settings having the highest burden of *Shigella* morbidity and mortality.

**Figure 3:**
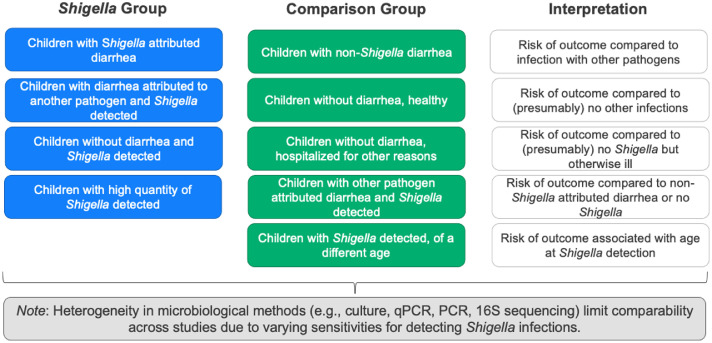
Examples of heterogeneity in comparison groups for outcome measurement. Abbreviations: qPCR, quantitative polymerase chain reaction.

Our ability to meta-analyze these data was limited by substantial heterogeneity in comparison groups between studies and in how outcomes were measured. To illustrate the variability in comparison groups, we've summarized in Figure 3 some common ways children with *Shigella* were defined (in blue) and possible comparison groups (in green). The interpretation of the results is dependent on the combination of the two groups and may or may not be comparable between studies. In practice, it may not be possible to distinguish between the first two blue boxes (whether diarrhea is attributed to *Shigella* or to another pathogen) particularly in studies that do not test for multiple pathogens. Moreover, it was common for studies to not have any comparison group (particularly for diarrhea outcomes), which limits our ability to make conclusions regarding *Shigella* consequences, relative to other pathogens or to absence of diarrhea. The comparability of findings was further limited by study heterogeneity in pathogen confirmation techniques (e.g., varying sensitivity of polymerase chain reaction vs culture), assessment of costs, and adjustment for co-infections and confounding factors, including antibiotic use and differences in the standard of care over time and by setting. Furthermore, host factors, such as age, malnutrition, HIV, and measles, are all established risk factors for poor *Shigella* outcomes and although these characteristics were included in our descriptions of the studies, without a formal meta-analysis, we were unable to statistically assess their contribution to outcomes.

The strengths of this systematic review include the wide time span of the reviewed literature and extensive breadth of the outcomes assessed. This review identified several evidence gaps, including lack of data on neurodevelopmental outcomes, relatively short follow-up periods for shigellosis, and limited geographic diversity in study locations. Future trials of *Shigella*-specific vaccines or treatments with long-term follow-up will ultimately be best positioned to document *Shigella* consequences.

## Declaration of interests

The authors have no competing interests to declare.

## Appendix Supplementary materials

Image, application 1
