# Supplementary material for: Consequences of Shigella infection in young children: a systematic review
Source: Int J Infect Dis. 2023 Apr;129:78–95. doi: 10.1016/j.ijid.2023.01.034 (PMC10017352; doi:10.1016/j.ijid.2023.01.034)
Supplement: Supplementary file 1 [file mmc1.docx]

**Supplementary Materials**

[Appendix 1. Search strings 2](#_Toc123987201)

[**PubMed Search Terms:** 2](#_Toc123987202)

[**Embase Search Terms:** 2](#_Toc123987203)

[Appendix 2. Quality reporting form for studies included in systematic review 4](#_Toc123987204)

[Appendix 3. Reasons for exclusion at full-text review (n=316) 5](#_Toc123987205)

[Appendix 4. Included studies where the number of children with *Shigella* was not specified 19](#_Toc123987206)

[Appendix 5. Quality scores of included studies 21](#_Toc123987207)

[Appendix 6. Summary of other anthropometric measures 23](#_Toc123987208)

[Appendix 7. Summary of additional outcomes 24](#_Toc123987209)

# **Appendix 1. Search strings**

## **PubMed Search Terms:**

(shigella[MeSH] OR "Dysentery, Bacillary"[Mesh] OR shigella[Title/Abstract] OR shigellosis[Title/Abstract] OR shigell*[Title/Abstract] OR dysentery[Title/Abstract] OR "bloody diarrhea"[Title/Abstract] OR "bloody stool"[Title/Abstract]) AND (child*[all fields] OR infant*[all fields] OR newborn[all fields] OR baby[all fields] OR babies[all fields] OR neonat*[all fields] OR pediatric[all fields] OR adoles*[all fields] OR teen*[all fields] OR youth*[all fields]) NOT ("editorial"[Publication Type] OR "letter"[Publication Type] OR "review"[Publication Type] OR systematic[sb]) AND ("1980/01/01"[Date - Publication] : "3000"[Date - Publication]) AND (((Deprived Countries[all fields] OR Deprived Population[all fields] OR Deprived Populations[all fields] OR Developing Countries[all fields] OR Developing Country[all fields] OR Developing Economies[all fields] OR Developing Economy[all fields] OR Developing Nation[all fields] OR Developing Nations[all fields] OR Developing Population[all fields] OR Developing Populations[all fields] OR Developing World[all fields] OR LAMI Countries[all fields] OR LAMI Country[all fields] OR Less Developed Countries[all fields] OR Less Developed Country[all fields] OR Less Developed Economies [all fields] OR Less Developed Nation[all fields] OR Less Developed Nations[all fields] OR Less Developed World[all fields] OR Lesser Developed Countries[all fields] OR Lesser Developed Nations[all fields] OR LMIC[all fields] OR LMICS[all fields] OR Low GDP[all fields] OR Low GNP[all fields] OR Low Gross Domestic[all fields] OR Low Gross National[all fields] OR Low Income Countries[all fields] OR Low Income Country[all fields] OR Low Income Economies [all fields] OR Low Income Economy[all fields] OR Low Income Nations[all fields] OR Low Income Population[all fields] OR Low Income Populations[all fields] OR Lower GDP[all fields] OR lower gross domestic[all fields] OR Lower Income Countries[all fields] OR Lower Income Country[all fields] OR Lower Income Nations[all fields] OR Lower Income Population[all fields] OR Lower Income Populations[all fields] OR Middle Income Countries[all fields] OR Middle Income Country[all fields] OR Middle Income Economies [all fields] OR Middle Income Nation[all fields] OR Middle Income Nations[all fields] OR Middle Income Population[all fields] OR Middle Income Populations[all fields] OR Poor Countries[all fields] OR Poor Country[all fields] OR Poor Economies [all fields] OR Poor Economy[all fields] OR Poor Nation[all fields] OR Poor Nations[all fields] OR Poor Population[all fields] OR Poor Populations[all fields] OR poor world[all fields] OR Poorer Countries[all fields] OR Poorer Economies [all fields] OR Poorer Economy[all fields] OR Poorer Nations[all fields] OR Poorer Population[all fields] OR Poorer Populations[all fields] OR Third World[all fields] OR Transitional Countries[all fields] OR Transitional Country[all fields] OR Transitional Economies[all fields] OR Transitional Economy[all fields] OR Under Developed Countries[all fields] OR Under Developed Country[all fields] OR under developed nations[all fields] OR Under Developed World[all fields] OR Under Served Population[all fields] OR Under Served Populations[all fields] OR Underdeveloped Countries[all fields] OR Underdeveloped Country[all fields] OR underdeveloped economies[all fields] OR underdeveloped nations[all fields] OR underdeveloped population[all fields] OR Underdeveloped World[all fields] OR Underserved Countries[all fields] OR Underserved Nations[all fields] OR Underserved Population[all fields] OR Underserved Populations[all fields]) OR (Afghanistan[tw] OR Albania[tw] OR Algeria[tw] OR "American Samoa"[tw] OR Angola[tw] OR Argentina[tw] OR "Argentine Republic"[tw] OR Armenia[tw] OR Azerbaijan[tw] OR Bangladesh[tw] OR Belarus[tw] OR Byelarus[tw] OR Belorussia[tw] OR Belize[tw] OR Benin[tw] OR Bhutan[tw] OR Bolivia[tw] OR Bosnia[tw] OR Botswana[tw] OR Brazil[tw] OR Bulgaria[tw] OR Burma[tw] OR "Burkina Faso"[tw] OR Burundi[tw] OR "Cabo Verde"[tw] OR "Cape verde"[tw] OR Cambodia[tw] OR Cameroon[tw] OR "Central African Republic"[tw] OR Chad[tw] OR China[tw] OR Colombia[tw] OR Comoros[tw] OR Comores[tw] OR Comoro[tw] OR Congo[tw] OR "Costa Rica"[tw] OR "Côte d'Ivoire"[tw] OR Cuba[tw] OR Djibouti[tw] OR Dominica[tw] OR "Dominican Republic"[tw] OR Ecuador[tw] OR Egypt[tw] OR "El Salvador"[tw] OR Eritrea[tw] OR Ethiopia[tw] OR Fiji[tw] OR Gabon[tw] OR Gambia[tw] OR Gaza[tw] OR "Georgia Republic"[tw] OR Georgian[tw] OR Ghana[tw] OR Grenada[tw] OR Grenadines[tw] OR Guatemala[tw] OR Guinea[tw] OR "Guinea Bissau"[tw] OR Guyana[tw] OR Haiti[tw] OR Herzegovina[tw] OR Hercegovina[tw] OR Honduras[tw] OR India[tw] OR Indonesia[tw] OR Iran[tw] OR Iraq[tw] OR Jamaica[tw] OR Jordan[tw] OR Kazakhstan[tw] OR Kenya[tw] OR Kiribati[tw] OR Korea[tw] OR Kosovo[tw] OR Kyrgyz[tw] OR Kirghizia[tw] OR Kirghiz[tw] OR Kirgizstan[tw] OR Kyrgyzstan[tw] OR "Lao PDR"[tw] OR Laos[tw] OR Lebanon[tw] OR Lesotho[tw] OR Liberia[tw] OR Libya[tw] OR Macedonia[tw] OR Madagascar[tw] OR Malawi[tw] OR Malay[tw] OR Malaya[tw] OR Malaysia[tw] OR Maldives[tw] OR Mali[tw] OR "Marshall Islands"[tw] OR Mauritania[tw] OR Mauritius[tw] OR Mexico[tw] OR Micronesia[tw] OR Moldova[tw] OR Mongolia[tw] OR Montenegro[tw] OR Morocco[tw] OR Mozambique[tw] OR Myanmar[tw] OR Namibia[tw] OR Nauru[tw] OR Nepal[tw] OR Nicaragua[tw] OR Niger[tw] OR Nigeria [tw] OR Pakistan [tw] OR Palau[tw] OR Panama[tw] OR "Papua New Guinea"[tw] OR Paraguay[tw] OR Peru [tw] OR Philippines[tw] OR Phillippines[tw] OR Philipines[tw] OR Phillipines[tw] OR Principe[tw] OR Romania[tw] OR Rwanda[tw] OR Ruanda[tw] OR Samoa[tw] OR "Sao Tome"[tw] OR Senegal[tw] OR Serbia[tw] OR "Sierra Leone"[tw] OR "Solomon Islands"[tw] OR Somalia[tw] OR "South Africa"[tw] OR "South Sudan"[tw] OR "Sri Lanka"[tw] OR "St Lucia"[tw] OR "St Vincent"[tw] OR Sudan[tw] OR Surinam[tw] OR Suriname[tw] OR Swaziland[tw] OR "Eswatini"[tw] OR Syria[tw] OR "Syrian Arab Republic"[tw] OR Tajikistan[tw] OR Tadzhikistan[tw] OR Tadjikistan[tw] OR Tadzhik[tw] OR Tanzania[tw] OR Thailand[tw] OR Timor[tw] OR Togo[tw] OR Tonga[tw] OR Tunisia[tw] OR Turkey[tw] OR Turkmen[tw] OR Turkmenistan[tw] OR Tuvalu[tw] OR Uganda[tw] OR Ukraine[tw] OR Uzbek[tw] OR Uzbekistan[tw] OR Vanuatu[tw] OR Venezuela[tw] OR Vietnam[tw] OR "West Bank"[tw] OR Yemen[tw] OR Zambia[tw] OR Zimbabwe[tw]))))

## **Embase Search Terms:**

('shigellosis'/exp OR 'shigella'/exp OR 'bloody diarrhea'/exp OR 'shigella':ab,ti OR 'shigellosis':ab,ti OR 'shigell*':ab,ti OR 'dysentery':ab,ti OR 'bloody diarrhea':ab,ti OR 'bloody stool':ab,ti) AND ('child*' OR 'infant*' OR 'newborn' OR 'baby' OR 'babies' OR 'neonat' OR 'pediatric' OR 'adoles*' OR 'teen*' OR 'youth') AND [1980-2022]/py NOT ([editorial]/lim OR [letter]/lim OR [note]/lim OR [review]/lim) AND ('deprived countries' OR 'deprived country' OR 'deprived nation' OR 'deprived nations' OR 'deprived population' OR 'deprived populations' OR 'deprived world' OR 'developing countries' OR 'developing country' OR 'developing economies' OR 'developing economy' OR 'developing nation' OR 'developing nations' OR 'developing population' OR 'developing populations' OR 'developing world' OR 'lami countries' OR 'lami country' OR 'less developed countries' OR 'less developed country' OR 'less developed economies' OR 'less developed economy' OR 'less developed nation' OR 'less developed nations' OR 'less developed population' OR 'less developed populations' OR 'less developed world' OR 'lesser developed countries' OR 'lesser developed country' OR 'lesser developed economies' OR 'lesser developed economy' OR 'lesser developed nation' OR 'lesser developed nations' OR 'lesser developed population' OR 'lesser developed populations' OR 'lesser developed world' OR 'lmic' OR 'lmics' OR 'low gdp' OR 'low gnp' OR 'low gross domestic' OR 'low gross national' OR 'low income countries' OR 'low income country' OR 'low income economies' OR 'low income economy' OR 'low income nation' OR 'low income nations' OR 'low income population' OR 'low income populations' OR 'lower gdp' OR 'lower gnp' OR 'lower gross domestic' OR 'lower gross national' OR 'lower income countries' OR 'lower income country' OR 'lower income economies' OR 'lower income economy' OR 'lower income nation' OR 'lower income nations' OR 'lower income population' OR 'lower income populations' OR 'middle income countries' OR 'middle income country' OR 'middle income economies' OR 'middle income economy' OR 'middle income nation' OR 'middle income nations' OR 'middle income population' OR 'middle income populations' OR 'poor countries' OR 'poor country' OR 'poor economies' OR 'poor economy' OR 'poor nation' OR 'poor nations' OR 'poor population' OR 'poor populations' OR 'poor world' OR 'poorer countries' OR 'poorer country' OR 'poorer economies' OR 'poorer economy' OR 'poorer nation' OR 'poorer nations' OR 'poorer population' OR 'poorer populations' OR 'poorer world' OR 'third world' OR 'transitional countries' OR 'transitional country' OR 'transitional economies' OR 'transitional economy' OR 'under developed countries' OR 'under developed country' OR 'under developed economies' OR 'under developed economy' OR 'under developed nation' OR 'under developed nations' OR 'under developed population' OR 'under developed populations' OR 'under developed world' OR 'under served countries' OR 'under served country' OR 'under served nation' OR 'under served nations' OR 'under served population' OR 'under served populations' OR 'under served world' OR 'underdeveloped countries' OR 'underdeveloped country' OR 'underdeveloped economies' OR 'underdeveloped economy' OR 'underdeveloped nation' OR 'underdeveloped nations' OR 'underdeveloped population' OR 'underdeveloped populations' OR 'underdeveloped world' OR 'underserved countries' OR 'underserved country' OR 'underserved nation' OR 'underserved nations' OR 'underserved population' OR 'underserved populations' OR 'underserved world' OR afghanistan:de,ti,ab OR albania:de,ti,ab OR algeria:de,ti,ab OR 'american samoa':de,ti,ab OR angola:de,ti,ab OR argentina:de,ti,ab OR 'argentine republic':de,ti,ab OR armenia:de,ti,ab OR azerbaijan:de,ti,ab OR bangladesh:de,ti,ab OR belarus:de,ti,ab OR byelarus:de,ti,ab OR belorussia:de,ti,ab OR belize:de,ti,ab OR benin:de,ti,ab OR bhutan:de,ti,ab OR bolivia:de,ti,ab OR bosnia:de,ti,ab OR botswana:de,ti,ab OR brazil:de,ti,ab OR bulgaria:de,ti,ab OR burma:de,ti,ab OR 'burkina faso':de,ti,ab OR burundi:de,ti,ab OR 'cabo verde':de,ti,ab OR 'cape verde':de,ti,ab OR cambodia:de,ti,ab OR cameroon:de,ti,ab OR 'central african republic':de,ti,ab OR chad:de,ti,ab OR china:de,ti,ab OR colombia:de,ti,ab OR comoros:de,ti,ab OR comores:de,ti,ab OR comoro:de,ti,ab OR congo:de,ti,ab OR 'costa rica':de,ti,ab OR 'cote d ivoire':de,ti,ab OR cuba:de,ti,ab OR djibouti:de,ti,ab OR dominica:de,ti,ab OR 'dominican republic':de,ti,ab OR ecuador:de,ti,ab OR egypt:de,ti,ab OR 'el salvador':de,ti,ab OR eritrea:de,ti,ab OR eswatini:de,ti,ab OR ethiopia:de,ti,ab OR fiji:de,ti,ab OR gabon:de,ti,ab OR gambia:de,ti,ab OR gaza:de,ti,ab OR 'georgia republic':de,ti,ab OR georgian:de,ti,ab OR ghana:de,ti,ab OR grenada:de,ti,ab OR grenadines:de,ti,ab OR guatemala:de,ti,ab OR guinea:de,ti,ab OR 'guinea bissau':de,ti,ab OR guyana:de,ti,ab OR haiti:de,ti,ab OR herzegovina:de,ti,ab OR hercegovina:de,ti,ab OR honduras:de,ti,ab OR india:de,ti,ab OR indonesia:de,ti,ab OR iran:de,ti,ab OR iraq:de,ti,ab OR jamaica:de,ti,ab OR jordan:de,ti,ab OR kazakhstan:de,ti,ab OR kenya:de,ti,ab OR kiribati:de,ti,ab OR korea:de,ti,ab OR kosovo:de,ti,ab OR kyrgyz:de,ti,ab OR kirghizia:de,ti,ab OR kirghiz:de,ti,ab OR kirgizstan:de,ti,ab OR kyrgyzstan:de,ti,ab OR 'lao pdr':de,ti,ab OR laos:de,ti,ab OR lebanon:de,ti,ab OR lesotho:de,ti,ab OR liberia:de,ti,ab OR libya:de,ti,ab OR macedonia:de,ti,ab OR madagascar:de,ti,ab OR malawi:de,ti,ab OR malay:de,ti,ab OR malaya:de,ti,ab OR malaysia:de,ti,ab OR maldives:de,ti,ab OR mali:de,ti,ab OR 'marshall islands':de,ti,ab OR mauritania:de,ti,ab OR mauritius:de,ti,ab OR mexico:de,ti,ab OR micronesia:de,ti,ab OR moldova:de,ti,ab OR mongolia:de,ti,ab OR montenegro:de,ti,ab OR morocco:de,ti,ab OR mozambique:de,ti,ab OR myanmar:de,ti,ab OR namibia:de,ti,ab OR nauru:de,ti,ab OR nepal:de,ti,ab OR nicaragua:de,ti,ab OR niger:de,ti,ab OR nigeria:de,ti,ab OR pakistan:de,ti,ab OR palau:de,ti,ab OR panama:de,ti,ab OR 'papua new guinea':de,ti,ab OR paraguay:de,ti,ab OR peru:de,ti,ab OR philippines:de,ti,ab OR phillippines:de,ti,ab OR philipines:de,ti,ab OR phillipines:de,ti,ab OR principe:de,ti,ab OR romania:de,ti,ab OR rwanda:de,ti,ab OR ruanda:de,ti,ab OR samoa:de,ti,ab OR 'sao tome':de,ti,ab OR senegal:de,ti,ab OR serbia:de,ti,ab OR 'sierra leone':de,ti,ab OR 'solomon islands':de,ti,ab OR somalia:de,ti,ab OR 'south africa':de,ti,ab OR 'south sudan':de,ti,ab OR 'sri lanka':de,ti,ab OR 'st lucia':de,ti,ab OR 'st vincent':de,ti,ab OR sudan:de,ti,ab OR surinam:de,ti,ab OR suriname:de,ti,ab OR swaziland:de,ti,ab OR syria:de,ti,ab OR 'syrian arab republic':de,ti,ab OR tajikistan:de,ti,ab OR tadzhikistan:de,ti,ab OR tadjikistan:de,ti,ab OR tadzhik:de,ti,ab OR tanzania:de,ti,ab OR thailand:de,ti,ab OR timor:de,ti,ab OR togo:de,ti,ab OR tonga:de,ti,ab OR tunisia:de,ti,ab OR turkey:de,ti,ab OR turkmen:de,ti,ab OR turkmenistan:de,ti,ab OR tuvalu:de,ti,ab OR uganda:de,ti,ab OR ukraine:de,ti,ab OR uzbek:de,ti,ab OR uzbekistan:de,ti,ab OR vanuatu:de,ti,ab OR venezuela:de,ti,ab OR vietnam:de,ti,ab OR 'west bank':de,ti,ab OR yemen:de,ti,ab OR zambia:de,ti,ab OR zimbabwe:de,ti,ab) AND [embase]/lim NOT ([embase]/lim AND [medline]/lim)

# **Appendix 2. Quality reporting form for studies included in systematic review**

| **Method heading** | **Description** | **Score** |
| --- | --- | --- |
| **STROBE Guidelines** | |  |
| **Study design** | Present key elements of study design early in the paper. STROBE guidelines encourage the researcher to describe the actual methodology rather than using the words like “prospective” or “retrospective.” | 1 |
| **Setting** | Describe the setting, locations, and relevant dates, including periods of recruitment, exposure, follow-up, and data collection | 1 |
| **Participants** | (*a*) *Cohort study*—Give the eligibility criteria, and the sources and methods of selection of participants. Describe methods of follow-up  *Case-control study*—Give the eligibility criteria, and the sources and methods of case ascertainment and control selection. Give the rationale for the choice of cases and controls  *Cross-sectional study*—Give the eligibility criteria, and the sources and methods of selection of participants | 1 |
|  | (*b*) *Cohort study*—For matched studies, give matching criteria and number of exposed and unexposed  *Case-control study*—For matched studies, give matching criteria and the number of controls per case |  |
| **Variables** | Clearly define all outcomes, exposures, predictors, potential confounders, and effect modifiers. Give diagnostic criteria, if applicable | 1 |
| **Data sources/** **measurement** | For each variable of interest, give sources of data and details of methods of assessment (measurement). Describe comparability of assessment methods if there is more than one group | 1 |
| **Bias** | Describe any efforts to address potential sources of bias | 1 |
| **Study size** | Explain how the study size was arrived at. Per STROBE guidelines, “The method used to establish the study size needs to be reported along with the confidence intervals considered. This is essential for the reader to ascertain whether sufficient statistical precision has been attained in the study.” | 1 |
| **Quantitative** **variables** | Explain how quantitative variables were handled in the analyses. If applicable, describe which groupings were chosen and why | 1 |
| **Statistical** **methods** | (*a*) Describe all statistical methods, including those used to control for confounding | 1 |
|  | (*b*) Describe any methods used to examine subgroups and interactions |  |
|  | (*c*) Explain how missing data were addressed |  |
|  | (*d*) *Cohort study*—If applicable, explain how loss to follow-up was addressed  *Case-control study*—If applicable, explain how matching of cases and controls was addressed  *Cross-sectional study*—If applicable, describe analytical methods taking account of sampling strategy | 1 |
|  | (*e*) Describe any sensitivity analyses |  |

Abbreviations: STROBE, Strengthening the Reporting of Observational Studies in Epidemiology. Quality reporting form adapted from STROBE statement (von Elm et al., 2007) as implemented previously in a systematic review by Ganguly et al. (2015).

# **Appendix 3. Reasons for exclusion at full-text review (n=316)**

| **Study** | **Title** | **Journal** | **Exclusion Reason** |
| --- | --- | --- | --- |
| Afroze 2017 | Risk factors and outcome of Shigella encephalopathy in Bangladeshi children. | PLoS neglected tropical diseases | No outcomes reported among <5 yr except mortality |
| Afroze 2020 | Pathogen-specific risk of seizure in children with moderate-to-severe diarrhoea: Case control study with follow-up. | Tropical medicine & international health : TM & IH | No Shigella-specific outcomes |
| Agtini 2007 | Erratum: The burden of diarrhoea, shigellosis, and cholera in North Jakarta, Indonesia: Findings from 24 months surveillance (BMC Infectious Diseases) | BMC Infect. Dis. | No follow-up/cross-sectional |
| Ahmed 1993 | Initiation of food supplements and stopping of breast-feeding as determinants of weanling shigellosis. | Bulletin of the World Health Organization | No follow-up/cross-sectional |
| Ahmed 1994 | Family latrines and paediatric shigellosis in rural Bangladesh: benefit or risk? | International journal of epidemiology | No follow-up/cross-sectional |
| Ahmed 1997 | Epidemiology of shigellosis among children exposed to cases of Shigella dysentery: a multivariate assessment. | The American journal of tropical medicine and hygiene | No follow-up/cross-sectional |
| Akpede 1994 | Localized extracranial infections in children with acute bacterial meningitis. | Journal of tropical pediatrics | Shigella not detected/No testing |
| Alam 2000 | Association between clinical type of diarrhoea and growth of children under 5 years in rural Bangladesh. | International journal of epidemiology | Shigella not detected/No testing |
| Almatti 2012 | The role of rotavirus in acute gastroenteritis a study in the islamic hospital/amman/jordan | Arch. Dis. Child. | No Shigella-specific outcomes |
| Anand 1986 | Rectal histology in acute bacillary dysentery. | Gastroenterology | Doesn't include children <5 years |
| Aragón 1995 | Shigellosis in Mozambique: the 1993 outbreak rehabilitation—a follow-up study. | Tropical doctor | No outcomes reported among <5 yr except mortality |
| Armin 2007 | Which cases of gastroenteritis will tend to get into convulsion? | Pak. J. Med. Sci. | No follow-up/cross-sectional |
| Arthur 1992 | Diarrheal disease in Cambodian children at a camp in Thailand. | American journal of epidemiology | No Shigella-specific outcomes |
| Attia 2016 | Mortality in children with complicated severe acute malnutrition is related to intestinal and systemic inflammation: an observational cohort study. | The American journal of clinical nutrition | No follow-up/cross-sectional |
| Aziz 1990 | Reduction in diarrhoeal diseases in children in rural Bangladesh by environmental and behavioural modifications. | Transactions of the Royal Society of Tropical Medicine and Hygiene | Shigella not detected/No testing |
| Bahl 1997 | Experience with diarrhea training and treatment unit in Shimla. | Indian pediatrics | Shigella not detected/No testing |
| Baqui 1988 | Epidemiological and clinical characteristics of shigellosis in rural Bangladesh. | Journal of diarrhoeal diseases research | Outcomes not stratified among <5 yr |
| Baranwal 2009 | Diarrhea associated hemolytic uremic syndrome: a 3-year PICU experience from Nepal. | Indian journal of pediatrics | No Shigella-specific outcomes |
| Basu 2007 | Effect of Lactobacillus rhamnosus GG in persistent diarrhea in Indian children: a randomized controlled trial. | Journal of clinical gastroenterology | Outcomes not stratified among <5 yr |
| Basualdo 2003 | Randomized comparison of azithromycin versus cefixime for treatment of shigellosis in children. | The Pediatric infectious disease journal | Outcomes not stratified among <5 yr |
| Begue 1994 | Diarrheal disease in Peru after the introduction of cholera. | The American journal of tropical medicine and hygiene | Doesn't include children <5 years |
| Benítez 1991 | [Etiology of bloody diarrhea in children from a rural community]. | Boletin medico del Hospital Infantil de Mexico | No Shigella-specific outcomes |
| Benmessaoud 2015 | Aetiology, epidemiology and clinical characteristics of acute moderate-to-severe diarrhoea in children under 5 years of age hospitalized in a referral paediatric hospital in Rabat, Morocco. | Journal of medical microbiology | No Shigella-specific outcomes |
| Bennish 1990 | Death in shigellosis: incidence and risk factors in hospitalized patients. | The Journal of infectious diseases | No follow-up/cross-sectional |
| Bennish 1991 | Intestinal obstruction during shigellosis: incidence, clinical features, risk factors, and outcome. | Gastroenterology | Outcomes not stratified among <5 yr |
| Bhan 1989 | Descriptive epidemiology of persistent diarrhoea among young children in rural northern India. | Bulletin of the World Health Organization | Fewer than 5 children with shigella |
| Bhandari 1992 | Mortality associated with acute watery diarrhea, dysentery and persistent diarrhea in rural North India. | Acta paediatrica | Shigella not detected/No testing |
| Bhimma 1997 | Post-dysenteric hemolytic uremic syndrome in children during an epidemic of Shigella dysentery in Kwazulu/Natal. | Pediatric nephrology (Berlin, Germany) | No Shigella-specific outcomes |
| Bhimma 2001 | Re-evaluating criteria for peritoneal dialysis in "classical" (D+) hemolytic uremic syndrome. | Clinical nephrology | No Shigella-specific outcomes |
| Black 1985 | Gastroenteritis caused by Escherichia coli and Shigella retards the growth of children | Nutritional reviews | Duplicate data |
| Bowen-Jones 1989 | Infection and cross-infection in a paediatric gastro-enteritis unit. | Curationis | No Shigella-specific outcomes |
| Brander 2018 | Antibiotic management of moderate-to-severe diarrhea may reduce risk of linear growth faltering in children: A secondary analysis of gems cases | Am. J. Trop. Med. Hyg. | Shigella not detected/No testing |
| Brennhofer 2022 | Antibiotic use attributable to specific aetiologies of diarrhoea in children under 2 years of age in low-resource settings: a secondary analysis of the MAL-ED birth cohort. | BMJ Open | No follow-up/cross-sectional |
| Bukiy 2019 | PECULIARITIES OF CYTOKINE RESPONSE IN CHILDREN WITH SHIGELLOSIS AND CONCOMITANT CYTOMEGALOVIRUS INFECTION. | Georgian medical news | Outcomes not stratified among <5 yr |
| Buskirk 2022 | Mucosal Immune Profiles Associated with Diarrheal Disease Severity in Shigella- and Enteropathogenic Escherichia coli-Infected Children Enrolled in the Global Enteric Multicenter Study. | mBio | No follow-up/cross-sectional |
| Butler 1987 | Segmental necrotising enterocolitis: pathological and clinical features of 22 cases in Bangladesh. | Gut | No follow-up/cross-sectional |
| Butler 1987 | Risk factors for development of hemolytic uremic syndrome during shigellosis. | The Journal of pediatrics | No outcomes reported among <5 yr except mortality |
| Butler 1989 | Depletion of hepatic glycogen in the hypoglycaemia of fatal childhood diarrhoeal illnesses. | Transactions of the Royal Society of Tropical Medicine and Hygiene | No follow-up/cross-sectional |
| Butler 1989 | Causes of death and the histopathologic findings in fatal shigellosis. | The Pediatric infectious disease journal | Outcomes not stratified among <5 yr |
| Calva 1996 | Antimicrobial resistance in fecal flora: longitudinal community-based surveillance of children from urban Mexico. | Antimicrobial agents and chemotherapy | Fewer than 5 children with shigella |
| Chandyo 2010 | Two weeks of zinc administration to Nepalese children with pneumonia does not reduce the incidence of pneumonia or diarrhea during the next six months. | The Journal of nutrition | Shigella not detected/No testing |
| Chatterjee 1985 | The so-called epidemic of Shigella dysentery in West Bengal. | Tropical gastroenterology : official journal of the Digestive Diseases Foundation | Outcomes not stratified among <5 yr |
| Chisti 2010 | Characteristics of children with Shigella encephalopathy: experience from a large urban diarrhea treatment center in Bangladesh. | The Pediatric infectious disease journal | No follow-up/cross-sectional |
| Chokoshvili 2018 | CHARACTERISTICS OF DIARRHEAL DISEASE COMPLICATED WITH HEMOLYTIC UREMIC SYNDROME AMONG CHILDREN IN GEORGIA, 2009-2016. | Georgian medical news | No follow-up/cross-sectional |
| Chopra 1997 | Epidemic shigella dysentery in children in northern KwaZulu-Natal. | South African medical journal = Suid-Afrikaanse tydskrif vir geneeskunde | No Shigella-specific outcomes |
| Clarkson 2020 | Immune Response Characterization after Controlled Infection with Lyophilized Shigella sonnei 53G. | mSphere | Study not in LMIC |
| Cravioto 1988 | Prospective study of diarrhoeal disease in a cohort of rural Mexican children: incidence and isolated pathogens during the first two years of life. | Epidemiology and infection | No follow-up/cross-sectional |
| Cruz 1995 | Fecal excretion of leukotriene C4 during human disease due to Shigella dysenteriae. | Journal of pediatric gastroenterology and nutrition | No follow-up/cross-sectional |
| Cuteanu 1984 | Antidysentery vaccination by live vaccine Vadizen (Sh. flexneri T32-Istrati) in children collectivities in PH County. | Archives roumaines de pathologie experimentales et de microbiologie | Study not in LMIC |
| Das 2012 | Changing trend of persistent diarrhoea in young children over two decades: observations from a large diarrhoeal disease hospital in Bangladesh. | Acta paediatrica (Oslo, Norway : 1992) | No follow-up/cross-sectional |
| Das 2013 | Clinical characteristics, etiology and antimicrobial susceptibility among overweight and obese individuals with diarrhea: observed at a large diarrheal disease hospital, Bangladesh. | PloS one | No Shigella-specific outcomes |
| Datta 1990 | Effect of protein rich diet during acute phase of dysentery. | Indian journal of public health | No Shigella-specific outcomes |
| Davies 2008 | Shigella bacteraemia over a decade in Soweto, South Africa. | Transactions of the Royal Society of Tropical Medicine and Hygiene | No outcomes reported among <5 yr except mortality |
| deAndrade 1999 | [Lethality in hospitalized infants with acute diarrhea: risk factors associated with death]. | Revista da Associacao Medica Brasileira (1992) | No follow-up/cross-sectional |
| Delahoy 2012 | Cryptosporidium infection in children less than five years old with moderate-to-severe diarrhea in rural Western Kenya, 2008-2011 | Am. J. Trop. Med. Hyg. | No Shigella-specific outcomes |
| Donald 1987 | Shigellosis in the south-western Cape of Good Hope 1968-85. | Epidemiology and infection | No outcomes reported among <5 yr except mortality |
| Dosunmu-Ogunbi 1983 | Local pattern of acute enteric bacterial infections in man‚ÄìLagos, Nigeria. | Developments in biological standardization | No Shigella-specific outcomes |
| Dragomir 1984 | Investigations concerning the immunoprophylactic effect of the antidysentery vaccination by live vaccine Vadizen (Sh. flexneri T32-Istrati) in children. | Archives roumaines de pathologie experimentales et de microbiologie | Study not in LMIC |
| Duan 1988 | Acute diarrhea in children in Shanghai. | Kansenshogaku zasshi. The Journal of the Japanese Association for Infectious Diseases | No outcomes reported among <5 yr except mortality |
| Duong 2018 | No Clinical Benefit of Empirical Antimicrobial Therapy for Pediatric Diarrhea in a High-Usage, High-Resistance Setting. | Clinical infectious diseases : an official publication of the Infectious Diseases Society of America | No Shigella-specific outcomes |
| DuPont 1992 | Oral aztreonam, a poorly absorbed yet effective therapy for bacterial diarrhea in US travelers to Mexico. | JAMA | Doesn't include children <5 years |
| Dutta 1995 | Assessing the cause of in-patients pediatric diarrheal deaths: an analysis of hospital records. | Indian pediatrics | No follow-up/cross-sectional |
| Dutta 1995 | Comparative efficacy of furazolidone and nalidixic acid in the empirical treatment of acute invasive diarrhea: randomized clinical trial. | Indian pediatrics | Shigella not detected/No testing |
| Elfving 2016 | Acute Uncomplicated Febrile Illness in Children Aged 2-59 months in Zanzibar - Aetiologies, Antibiotic Treatment and Outcome. | PloS one | No Shigella-specific outcomes |
| El Nageh 1984 | Shigella dysentery in Tripoli, Libya. | The Journal of tropical medicine and hygiene | No follow-up/cross-sectional |
| Ercumen 2015 | Upgrading a piped water supply from intermittent to continuous delivery and association with waterborne illness: a matched cohort study in urban India. | PLoS medicine | Shigella not detected/No testing |
| Fagundes-Neto 1999 | Acute diarrhea and malnutrition: lethality risk in hospitalized infants. | Journal of the American College of Nutrition | No outcomes reported among <5 yr except mortality |
| Faruque 1998 | Shigellosis in children: a clinico-epidemiological comparison between Shigella dysenteriae type I and Shigella flexneri. | Annals of tropical paediatrics | No follow-up/cross-sectional |
| Faruque 2015 | Characteristics, etiology and nutritional consequences of moderate-to-severe diarrhea associated with convulsion among rural under-5 children | Trop. Med. Int. Health | No Shigella-specific outcomes |
| Fauveau 1991 | Diarrhoea mortality in rural Bangladeshi children. | Journal of tropical pediatrics | No outcomes reported among <5 yr except mortality |
| Ferreccio 1991 | Epidemiologic patterns of acute diarrhea and endemic Shigella infections in children in a poor periurban setting in Santiago, Chile. | American journal of epidemiology | Study not in LMIC |
| Gaensbauer 2017 | Efficacy of a novel nutritional product in acute childhood diarrhea in guatemala: Secondary and exploratory analyses of a randomized, double blind, placebo controlled trial | Open Forum Infect. Dis. | Shigella not detected/No testing |
| Garenne 2007 | Sex-specific responses to zinc supplementation in Nouna, Burkina Faso. | Journal of pediatric gastroenterology and nutrition | Shigella not detected/No testing |
| Gazi 2022 | Infection With Escherichia Coli Pathotypes Is Associated With Biomarkers of Gut Enteropathy and Nutritional Status Among Malnourished Children in Bangladesh. | Front Cell Infect Microbiol | No follow-up/cross-sectional |
| Gendrel 1984 | [Acute gastroenteritis and breast feeding in Gabon. Preliminary results]. | Medecine tropicale : revue du Corps de sante colonial | No follow-up/cross-sectional |
| Gendrel 1985 | [Etiology of acute infantile gastroenteritis in Gabon]. | Bulletin de la Societe de pathologie exotique et de ses filiales | Full text not available |
| Gendrel 1997 | One-dose treatment with pefloxacin for infection due to multidrug-resistant Shigella dysenteriae type 1 in Burundi. | Clinical infectious diseases : an official publication of the Infectious Diseases Society of America | Outcomes not stratified among <5 yr |
| George 2015 | Shigella Infections in Household Contacts of Pediatric Shigellosis Patients in Rural Bangladesh. | Emerging infectious diseases | No follow-up/cross-sectional |
| Gilman 1980 | Randomized trial of high- and low-dose ampicillin therapy for treatment of severe dysentery due to Shigella dysenteriae type 1. | Antimicrobial agents and chemotherapy | Outcomes not stratified among <5 yr |
| Gilman 1981 | Single-dose ampicillin therapy for severe shigellosis in Bangladesh. | The Journal of infectious diseases | Outcomes not stratified among <5 yr |
| Giugliano 1986 | Longitudinal study of diarrhoeal disease in a peri-urban community in Manaus (Amazon-Brazil). | Annals of tropical medicine and parasitology | No follow-up/cross-sectional |
| Goh Rowland 1985 | The etiology of diarrhoea studied in the community in young urban Gambian children. | Journal of diarrhoeal diseases research | No Shigella-specific outcomes |
| Gosselin 2017 | Etiology of Diarrhea, Nutritional Outcomes, and Novel Intestinal Biomarkers in Tanzanian Infants. | Journal of pediatric gastroenterology and nutrition | No Shigella-specific outcomes |
| Grant 1998 | Surgical lessons learned from the Shigella dysenteriae type I epidemic. | Journal of the Royal College of Surgeons of Edinburgh | No Shigella-specific outcomes |
| Grigoryan 2020 | The etiological structure of patients hospitalized in pediatric infectious diseases department of ‚ÄúNork‚Äù ICH during January- June 2018 | J. Infect. Public Health | No Shigella-specific outcomes |
| Guerin 1985 | Aetiological forms | Child Trop | No follow-up/cross-sectional |
| Guerin 2003 | Shigella dysenteriae serotype 1 in west Africa: intervention strategy for an outbreak in Sierra Leone. | Lancet (London, England) | No outcomes reported among <5 yr except mortality |
| Guerin 2004 | Case management of a multidrug-resistant Shigella dysenteriae serotype 1 outbreak in a crisis context in Sierra Leone, 1999-2000. | Transactions of the Royal Society of Tropical Medicine and Hygiene | No outcomes reported among <5 yr except mortality |
| Guerrant 1983 | Prospective study of diarrheal illnesses in northeastern Brazil: patterns of disease, nutritional impact, etiologies, and risk factors. | The Journal of infectious diseases | Outcomes not stratified among <5 yr |
| Guerrero 1994 | Asymptomatic Shigella infections in a cohort of Mexican children younger than two years of age. | The Pediatric infectious disease journal | No Shigella-specific outcomes |
| Gupta 1987 | Outbreak of dysentery due to Shigella flexneri type 2a. | The Journal of communicable diseases | No Shigella-specific outcomes |
| Gupta 1996 | Epidemiological and clinical profiles of acute invasive diarrhoea with special reference to mucoid episodes: a rural community-based longitudinal study. | Transactions of the Royal Society of Tropical Medicine and Hygiene | No Shigella-specific outcomes |
| Haider 2000 | Neonatal diarrhea in a diarrhea treatment center in Bangladesh: clinical presentation, breastfeeding management and outcome. | Indian pediatrics | No Shigella-specific outcomes |
| Han 1987 | An outbreak of dysentery due to Shigella dysenteriae type 1 in Rangoon, Burma. | Journal of diarrhoeal diseases research | No outcomes reported among <5 yr except mortality |
| Han 1990 | Measles-associated diarrhoea in the Infectious Diseases Hospital, Rangoon | J Trop Med Hyg | Fewer than 5 children with shigella |
| Haque 2003 | Epidemiologic and clinical characteristics of acute diarrhea with emphasis on Entamoeba histolytica infections in preschool children in an urban slum of Dhaka, Bangladesh. | The American journal of tropical medicine and hygiene | No Shigella-specific outcomes |
| Hegde 2013 | Burden of laboratory-confirmed shigellosis infections in guatemala 2007-2012: Results from a population-based surveillance system | Am. J. Trop. Med. Hyg. | Outcomes not stratified among <5 yr |
| Hegde 2019 | Burden of laboratory-confirmed shigellosis infections in Guatemala 2007-2012: results from a population-based surveillance system. | BMC public health | Outcomes not stratified among <5 yr |
| Helvaci 1998 | Comparative efficacy of cefixime and ampicillin-sulbactam in shigellosis in children. | Acta paediatrica Japonica : Overseas edition | Outcomes not stratified among <5 yr |
| Henry 1987 | Dysentery, not watery diarrhoea, is associated with stunting in Bangladeshi children. | Human nutrition. Clinical nutrition | Shigella not detected/No testing |
| Hong 2006 | [Clinical Characteristics of Extended-spectrum beta-lactamase Producing Shigella sonnei Infection Outbreaked in Chungju Area.]. | The Korean journal of laboratory medicine | Study not in LMIC |
| Hossain 1998 | Single dose vitamin A treatment in acute shigellosis in Bangladesh children: randomised double blind controlled trial. | BMJ (Clinical research ed.) | Outcomes not stratified among <5 yr |
| Hossain 2019 | Epidemiology and clinical presentation of cryptosporidium-associated diarrheal disease in children under five from three countries in subsaharan Africa | Am. J. Trop. Med. Hyg. | No Shigella-specific outcomes |
| Hossain 2022 | Prolonged diarrhea among under-five children in Bangladesh: Burden and risk factors. | PLoS One | No follow-up/cross-sectional |
| Hou 1985 | [Clinical efficacy of cefazolin made in China]. | Zhonghua nei ke za zhi | No Shigella-specific outcomes |
| Hovhannisyan 2019 | Rotavirus infection among vaccinated hospitalized children | Int. J. Infect. Dis. | No Shigella-specific outcomes |
| Huppertz 1986 | An epidemic of bacillary dysentery in western Rwanda 1981-1982. | The Central African journal of medicine | No Shigella-specific outcomes |
| Hurmuzache 1982 | [Convulsive syndrome in infantile shigellosis]. | Revista medico-chirurgicala a Societatii de Medici si Naturalisti din Iasi | Study not in LMIC |
| Iqbal 2015 | Association of enteric pathogens with poor growth in children in a rural district of Pakistan | Am. J. Trop. Med. Hyg. | Fewer than 5 children with shigella |
| Ise 1994 | Clinical evaluation and bacterial survey in infants and young children with diarrhoea in the Santa Cruz district, Bolivia. | Journal of tropical pediatrics | No Shigella-specific outcomes |
| Islam 1986 | Morbidity and mortality in a diarrhoeal diseases hospital in Bangladesh. | Transactions of the Royal Society of Tropical Medicine and Hygiene | No outcomes reported among <5 yr except mortality |
| Islam 1988 | A steep decline of death in a shigellosis epidemic in Bangladesh by a community‚Äìparticipated intervention. | Journal of diarrhoeal diseases research | No outcomes reported among <5 yr except mortality |
| Islam 1994 | Pathology of shigellosis and its complications. | Histopathology | No Shigella-specific outcomes |
| Jha 2007 | Clinico-laboratory profile of haemolytic uremic syndrome. | Kathmandu University medical journal (KUMJ) | No Shigella-specific outcomes |
| Johansen 1989 | Intestinal permeability assessed with polyethylene glycols in children with diarrhea due to rotavirus and common bacterial pathogens in a developing community. | Journal of pediatric gastroenterology and nutrition | No follow-up/cross-sectional |
| Joshi 1980 | A study of bacterial infantile diarrhea. | Indian journal of pediatrics | No Shigella-specific outcomes |
| Kabir 1992 | High-protein diet facilitates growth of children after shigellosis | Glimpse | Duplicate data |
| Kain 1991 | Etiology of childhood diarrhea in Beijing, China. | Journal of clinical microbiology | No follow-up/cross-sectional |
| Kamala 1996 | Management of diarrhea in a DTU. | Indian pediatrics | No Shigella-specific outcomes |
| Kamidani 2017 | Serial multiplex PCR may help differentiate causal pathogens from colonizing organisms in children with acute diarrhea in Guatemala | Open Forum Infect. Dis. | Shigella not detected/No testing |
| Karacan 2007 | Evaluation of shigellosis in a Turkish children's hospital. | Pediatrics international : official journal of the Japan Pediatric Society | No follow-up/cross-sectional |
| Keddy 2012 | Systemic shigellosis in South Africa. | Clinical infectious diseases : an official publication of the Infectious Diseases Society of America | Outcomes not stratified among <5 yr |
| Keenan 2020 | Cause-specific mortality of children younger than 5 years in communities receiving biannual mass azithromycin treatment in Niger: verbal autopsy results from a cluster-randomised controlled trial. | The Lancet. Global health | Shigella not detected/No testing |
| Kernéis 2009 | A look back at an ongoing problem: Shigella dysenteriae type 1 epidemics in refugee settings in Central Africa (1993-1995). | PloS one | Shigella not detected/No testing |
| Ketema 1997 | Persistent diarrhoea: socio-demographic and clinical profile of 264 children seen at a referral hospital in Addis Ababa. | Ethiopian medical journal | Shigella not detected/No testing |
| Khan 1980 | Contrasting epidemiology of shigellae dysenteriae and shigellae flexneri, Dacca. | Transactions of the Royal Society of Tropical Medicine and Hygiene | No Shigella-specific outcomes |
| Khan 1983 | Clinical illnesses and causes of death in a Burmese refugee camp in Bangladesh. | International journal of epidemiology | No Shigella-specific outcomes |
| Khan 1985 | Fourteen years of shigellosis in Dhaka: an epidemiological analysis. | International journal of epidemiology | No outcomes reported among <5 yr except mortality |
| Khan 1997 | Treatment of shigellosis: V. Comparison of azithromycin and ciprofloxacin. A double-blind, randomized, controlled trial. | Annals of internal medicine | Doesn't include children <5 years |
| Khan 1999 | Central nervous system manifestations of childhood shigellosis: prevalence, risk factors, and outcome. | Pediatrics | Outcomes not stratified among <5 yr |
| Khan 2006 | Detection of intra-familial transmission of shigella infection using conventional serotyping and pulsed-field gel electrophoresis. | Epidemiology and infection | No follow-up/cross-sectional |
| Khan 2013 | Gastrointestinal and extra-intestinal manifestations of childhood shigellosis in a region where all four species of Shigella are endemic. | PloS one | Outcomes not stratified among <5 yr |
| Khan 2020 | Electronic decision support and diarrhoeal disease guideline adherence (mHDM): a cluster randomised controlled trial | Lancet Digit. Heal. | Shigella not detected/No testing |
| Khatry 1995 | Epidemiology of xerophthalmia in Nepal. A pattern of household poverty, childhood illness, and mortality. The Sarlahi Study Group. | Archives of ophthalmology (Chicago, Ill. : 1960) | Shigella not detected/No testing |
| Khin-Maung-U 1987 | Clinical features, including haemolytic-uraemic syndrome, in Shigella dysenteriae type 1 infection in children of Rangoon. | Journal of diarrhoeal diseases research | Fewer than 5 children with shigella |
| Khiveh 2017 | Effects of rhubarb (Rheum ribes L.) syrup on dysenteric diarrhea in children: a randomized, double-blind, placebo-controlled trial. | Journal of integrative medicine | Outcomes not stratified among <5 yr |
| Khuffash 1988 | Acute gastroenteritis: clinical features according to etiologic agents. | Clinical pediatrics | Outcomes not stratified among <5 yr |
| Kim 2013 | Epidemiological Characteristics of Imported Shigellosis in Korea, 2010-2011 | Osong Public Health Res. Perspect. | Study not in LMIC |
| Kingamkono 1999 | Enteropathogenic bacteria in faecal swabs of young children fed on lactic acid-fermented cereal gruels. | Epidemiology and infection | No Shigella-specific outcomes |
| Knee 2021 | Effects of an urban sanitation intervention on childhood enteric infection and diarrhea in Maputo, Mozambique: A controlled before-and-after trial. | Elife | No Shigella-specific outcomes |
| Kosek 2008 | Epidemiology of highly endemic multiply antibiotic-resistant shigellosis in children in the Peruvian Amazon. | Pediatrics | No Shigella-specific outcomes |
| Kotloff 2013 | Burden and aetiology of diarrhoeal disease in infants and young children in developing countries (the Global Enteric Multicenter Study, GEMS): a prospective, case-control study. | Lancet (London, England) | No Shigella-specific outcomes |
| Kotloff 2019 | The incidence, aetiology, and adverse clinical consequences of less severe diarrhoeal episodes among infants and children residing in low-income and middle-income countries: a 12-month case-control study as a follow-on to the Global Enteric Multicenter S | The Lancet. Global health | No Shigella-specific outcomes |
| Kovitangkoon 1990 | Hemolytic uremic syndrome associated with Shigellosis: a report of 8 cases. | Journal of the Medical Association of Thailand = Chotmaihet thangphaet | Fewer than 5 children with shigella |
| Kuşkonmaz 2009 | Comparison of acute bloody and watery diarrhea: a case control study. | The Turkish journal of pediatrics | No Shigella-specific outcomes |
| Kunju 2019 | Etiology and short term outcome of acute febrile encephalopathy among children in a tertiary care centre in South India | J. Neurol. Sci. | No Shigella-specific outcomes |
| Lamabadusuriya 1991 | Changing antibiotic sensitivity patterns in shigellosis. | The Ceylon medical journal | Outcomes not stratified among <5 yr |
| Lamb 2017 | Nutritional status is not associated with diarrhea duration or weight recovery in young children in a resource-poor setting | Open Forum Infect. Dis. | No Shigella-specific outcomes |
| Lappan 2019 | Meta-taxonomic analysis of prokaryotic and eukaryotic gut flora in stool samples from visceral leishmaniasis cases and endemic controls in Bihar State India. | PLoS neglected tropical diseases | Doesn't include children <5 years |
| Laureillard 1998 | [Ciprofloxacin in the treatment of dysentery caused by type 1 Shigella dysenteriae during an epidemic in Rwandan refugees in Goma in 1994]. | Sante (Montrouge, France) | Shigella not detected/No testing |
| Le 2018 | Incidence and etiology of diarrhea in children: A two-year active surveillance in an urban community of vietnam | Am. J. Trop. Med. Hyg. | No Shigella-specific outcomes |
| Lee 2014 | Effects of Shigella-, Campylobacter- and ETEC-associated diarrhea on childhood growth. | The Pediatric infectious disease journal | Outcomes not stratified among <5 yr |
| Lee 2016 | A Comparison of Diarrheal Severity Scores in the MAL-ED Multisite Community-Based Cohort Study. | Journal of pediatric gastroenterology and nutrition | No Shigella-specific outcomes |
| Legros 1999 | Risk factors for death in hospitalized dysentery patients in Rwanda. | Tropical medicine & international health : TM & IH | Shigella not detected/No testing |
| Levine 2020 | Diarrhoeal disease and subsequent risk of death in infants and children residing in low-income and middle-income countries: analysis of the GEMS case-control study and 12-month GEMS-1A follow-on study. | The Lancet. Global health | No outcomes reported among <5 yr except mortality |
| Lexomboon 1994 | Control randomized study of rehydration/rehydration with dioctahedral smectite in ambulatory Thai infants with acute diarrhea. | The Southeast Asian journal of tropical medicine and public health | No Shigella-specific outcomes |
| Lindblad 2001 | Sodium and water homeostasis in children with shigellosis. | Acta paediatrica (Oslo, Norway : 1992) | No follow-up/cross-sectional |
| Lins 2000 | [Diarrheal disease in hospitalized children - importance of the persistent diarrhea]. | Jornal de pediatria | Shigella not detected/No testing |
| Lins 2003 | [Risk factors for persistent diarrhea in infants]. | Arquivos de gastroenterologia | Shigella not detected/No testing |
| Long 2013 | Associations between nutritional status, household factors and specific diarrheal pathogens among children in Mirzapur, Bangladesh | Ann. Nutr. Metab. | No follow-up/cross-sectional |
| Mølbak 1994 | The etiology of early childhood diarrhea: a community study from Guinea-Bissau. | The Journal of infectious diseases | No Shigella-specific outcomes |
| Mackenjee 1984 | Aetiology of diarrhoea in adequately nourished young African children in Durban, South Africa. | Annals of tropical paediatrics | No Shigella-specific outcomes |
| Mahalanabis 1991 | Prognostic indicators and risk factors for increased duration of acute diarrhoea and for persistent diarrhoea in children. | International journal of epidemiology | Shigella not detected/No testing |
| Mahbub 2012 | Analysis of Different Prognostic Indicators for Malnutrition and Shigella flexneri Infection Among the Children in Bangladesh. | Indian journal of microbiology | No follow-up/cross-sectional |
| Maldonado 1997 | Host and viral factors affecting the decreased immunogenicity of Sabin type 3 vaccine after administration of trivalent oral polio vaccine to rural Mayan children. | The Journal of infectious diseases | No Shigella-specific outcomes |
| Maldonado 1998 | Population-based prevalence of symptomatic and asymptomatic astrovirus infection in rural Mayan infants. | The Journal of infectious diseases | No Shigella-specific outcomes |
| Maleta 2020 | Infections and systemic inflammation are associated with lower plasma concentration of insulin-like growth factor I among Malawian children. | The American journal of clinical nutrition | No follow-up/cross-sectional |
| Mandomando 2011 | The aetiology of paediatric diarrhoea in Southern Africa (II) - The Global Enteric Multi-Center Study (GEMS): Laboratory aspects of Mozambique site | Trop. Med. Int. Health | No Shigella-specific outcomes |
| Maranhão 2008 | The epidemiological and clinical characteristics and nutritional development of infants with acute diarrhoea, in north-eastern Brazil. | Annals of tropical medicine and parasitology | No Shigella-specific outcomes |
| Marfin 1994 | Infectious disease surveillance during emergency relief to Bhutanese refugees in Nepal. | JAMA | No follow-up/cross-sectional |
| Mathan 1984 | Epidemic dysentery caused by the Shiga bacillus in a southern Indian village. | Journal of diarrhoeal diseases research | No Shigella-specific outcomes |
| Mattila 1993 | Short-term treatment of traveler's diarrhea with norfloxacin: a double-blind, placebo-controlled study during two seasons. | Clinical infectious diseases : an official publication of the Infectious Diseases Society of America | Doesn't include children <5 years |
| Mazumder 1997 | Reactive arthritis associated with Shigella dysenteriae type 1 infection. | Journal of diarrhoeal diseases research | Doesn't include children <5 years |
| Medeiros 2018 | Molecular characterization of virulence and antimicrobial resistance profile of Shigella species isolated from children with moderate to severe diarrhea in northeastern Brazil. | Diagnostic microbiology and infectious disease | No follow-up/cross-sectional |
| Meijman 1996 | [Physicians without borders and diarrhea as a cause of illness and death in refugee camps]. | Nederlands tijdschrift voor geneeskunde | No follow-up/cross-sectional |
| Mikhail 1989 | Microbiologic and clinical study of acute diarrhea in children in Aswan, Egypt. | Scandinavian journal of infectious diseases | No outcomes reported among <5 yr except mortality |
| Mitra 1990 | Fatal dysentery in rural Bangladesh. | Journal of diarrhoeal diseases research | No Shigella-specific outcomes |
| Mitra 1997 | Long-term oral supplementation with iron is not harmful for young children in a poor community of Bangladesh. | The Journal of nutrition | Shigella not detected/No testing |
| Moalla 1994 | [Etiology of acute diarrhea in children]. | La Tunisie medicale | No follow-up/cross-sectional |
| Mohebi 2021 | Evaluate the distribution of virulence genes and to investigate antibiotic resistance pattern among Shigella species isolated from children with shigellosis in Iran | Gene Rep. | No follow-up/cross-sectional |
| Moolasart 1999 | Comparison of the efficacy of ceftibuten and norfloxacin in the treatment of acute gastrointestinal infection in children. | The Southeast Asian journal of tropical medicine and public health | Outcomes not stratified among <5 yr |
| Moya-Alvarez 2021 | Vitamin C levels in a Central-African mother-infant cohort: Does hypovitaminosis C increase the risk of enteric infections? | Matern Child Nutr | No Shigella-specific outcomes |
| Moyo 2017 | Comprehensive Analysis of Prevalence, Epidemiologic Characteristics, and Clinical Characteristics of Monoinfection and Coinfection in Diarrheal Diseases in Children in Tanzania. | American journal of epidemiology | No follow-up/cross-sectional |
| Mudzamiri 1996 | Hospitalized dysentery cases during an outbreak of Shigella dysenteriae type I: Ndanga District Hospital, Zimbabwe. | The Central African journal of medicine | No Shigella-specific outcomes |
| Mutanda 1980 | Epidemiology of acute gastroenteritis in early childhood in Kenya. VI. Some clinical and laboratory characteristics relative to the aetiological agents. | East African medical journal | No follow-up/cross-sectional |
| Mutanda 1980 | Epidemiology of acute gastroenteritis in early childhood in Kenya: aetiological agents. | Tropical and geographical medicine | No follow-up/cross-sectional |
| Mutanda 1985 | Aetiology of diarrhoea in malnourished children at Kenyatta National Hospital. | East African medical journal | No follow-up/cross-sectional |
| Mwiru 2011 | Relationship of exclusive breast-feeding to infections and growth of Tanzanian children born to HIV-infected women. | Public health nutrition | Shigella not detected/No testing |
| Myaux 1996 | The effects of health services utilization on the recovery from dysentery. | Journal of tropical pediatrics | No Shigella-specific outcomes |
| Nahar 1988 | Prescribing for diarrheal diseases. | Indian pediatrics | No Shigella-specific outcomes |
| Nasrin 2022 | Incidence of Asymptomatic Shigella Infection and Association with the Composite Index of Anthropometric Failure among Children Aged 1-24 Months in Low-Resource Settings. | Life (Basel) | No follow-up/cross-sectional |
| Nathoo 1995 | Haemolytic uraemic syndrome following Shigella dysenteriae type 1 outbreak in Zimbabwe: a clinical experience. | The Central African journal of medicine | No Shigella-specific outcomes |
| Nathoo 1998 | Predictors of mortality in children hospitalized with dysentery in Harare, Zimbabwe. | The Central African journal of medicine | No Shigella-specific outcomes |
| Nezarieh 2015 | Distribution of virulence genes, enterotoxin and biofilm formation among enteroaggregative escherichia coli (EAEC) strains isolated from stools of children with diarrhea in South East Iran | Arch. Pediatr. Infect. Dis. | Shigella not detected/No testing |
| Nitiema 2011 | Burden of rotavirus and other enteropathogens among children with diarrhea in Burkina Faso. | International journal of infectious diseases : IJID : official publication of the International Society for Infectious Diseases | No follow-up/cross-sectional |
| Niyibitegeka 2021 | Economic burden of childhood diarrhea in Burundi. | Glob Health Res Policy | Shigella not detected/No testing |
| Niyogi 2001 | Changing patterns of serotypes and antimicrobial susceptibilities of Shigella species isolated from children in Calcutta, India. | Japanese journal of infectious diseases | No follow-up/cross-sectional |
| Niyogi 2003 | Multiresistant Shigella species isolated from childhood diarrhea cases in Kolkata, India. | Japanese journal of infectious diseases | No follow-up/cross-sectional |
| Nunes 2012 | Diarrhea associated with Shigella in children and susceptibility to antimicrobials. | Jornal de pediatria | No follow-up/cross-sectional |
| Nwanna-Nzewunwa 2016 | Epidemiology of childhood diarrheal diseases in the Niger-delta region of Nigeria: A retrospective study | Ann. of Global Health | Shigella not detected/No testing |
| O'Reilly 2012 | Risk factors for death among children less than 5 years old hospitalized with diarrhea in rural western Kenya, 2005-2007: a cohort study. | PLoS medicine | No outcomes reported among <5 yr except mortality |
| Oanã 1984 | Prophylaxis of bacillary dysentery by liver vaccine Vadizen (Sh. flexneri T32-Istrati) in children collectivities in IS County. | Archives roumaines de pathologie experimentales et de microbiologie | Shigella not detected/No testing |
| Oberhelman 1991 | Prospective study of systemic and mucosal immune responses in dysenteric patients to specific Shigella invasion plasmid antigens and lipopolysaccharides. | Infection and immunity | Outcomes not stratified among <5 yr |
| Odugbemi 1991 | Screening of children for enteric bacterial pathogens in the outborn neonatal ward in Lagos, Nigeria. | European journal of epidemiology | No follow-up/cross-sectional |
| Olotu 2008 | Haemolytic uraemic syndrome in children admitted to a rural district hospital in Kenya. | Tropical doctor | No follow-up/cross-sectional |
| Omore 2009 | Mortality among children with moderate-to-severe diarrhea in Rural Western Kenya, 2008 | Am. J. Trop. Med. Hyg. | No outcomes reported among <5 yr except mortality |
| Omore 2016 | Defining the burden and epidemiology of shigellosis in Rural Asembo, Western Kenya, 2007-2014 | Am. J. Trop. Med. Hyg. | No follow-up/cross-sectional |
| Oneko 2001 | Post-dysenteric hemolytic uremic syndrome in Bulawayo, Zimbabwe. | Pediatric nephrology (Berlin, Germany) | No Shigella-specific outcomes |
| Opintan 2010 | Pediatric diarrhea in southern Ghana: etiology and association with intestinal inflammation and malnutrition. | The American journal of tropical medicine and hygiene | No follow-up/cross-sectional |
| Paniagua 2007 | Two or more enteropathogens are associated with diarrhoea in Mexican children. | Annals of clinical microbiology and antimicrobials | No follow-up/cross-sectional |
| Paquet 1995 | [An outbreak of Shigella dysenteriae type 1 dysentery in a refugee camp in Rwanda]. | Sante (Montrouge, France) | No Shigella-specific outcomes |
| Patel 2009 | Zinc and copper supplementation in acute diarrhea in children: a double-blind randomized controlled trial. | BMC medicine | Shigella not detected/No testing |
| Patel 2012 | Risk factors for predicting diarrheal duration and morbidity in children with acute diarrhea. | Indian journal of pediatrics | Shigella not detected/No testing |
| Patwari 1995 | Persistent diarrhea: management in a diarrhea treatment unit. | Indian pediatrics | Fewer than 5 children with shigella |
| Pavlova 2020 | Shigellosis in children-clinical course and antimicrobial Sensitivity | Gen. Med. | No follow-up/cross-sectional |
| Penny 2004 | Randomized controlled trial of the effect of daily supplementation with zinc or multiple micronutrients on the morbidity, growth, and micronutrient status of young Peruvian children. | The American journal of clinical nutrition | Shigella not detected/No testing |
| Perin 2019 | A prospective cohort study investigating the relationship between the gut microbiota, environmental enteropathy and impaired growth in rural Bangladesh | Am. J. Trop. Med. Hyg | Duplicate data |
| Pernica 2015 | Rapid diagnostic testing and lactobacillus reuteri therapy for children with severe acute gastroenteritis in botswana: A pilot, factorial, randomized, controled, clinical trial | Can. J. Infect. Dis. Med. Microbiol. | No Shigella-specific outcomes |
| Pernica 2016 | Correlation of Clinical Outcomes With Multiplex Molecular Testing of Stool From Children Admitted to Hospital With Gastroenteritis in Botswana. | Journal of the Pediatric Infectious Diseases Society | Outcomes not stratified among <5 yr |
| Pernica 2017 | Rapid enteric testing to permit targeted antimicrobial therapy, with and without Lactobacillus reuteri probiotics, for paediatric acute diarrhoeal disease in Botswana: A pilot, randomized, factorial, controlled trial. | PloS one | No Shigella-specific outcomes |
| Pernica 2022 | Optimising the management of childhood acute diarrhoeal disease using a rapid test-and- treat strategy and/or Lactobacillus reuteri DSM 17938: a multicentre, randomised, controlled, factorial trial in Botswana. | BMJ Glob Health | No Shigella-specific outcomes |
| Pãnoiu 1984 | Economical evaluation of the dysentery vaccination in B Municipality. | Archives roumaines de pathologie experimentales et de microbiologie | No Shigella-specific outcomes |
| Phillips 1989 | Costs of treating diarrhoea in a children's hospital in Mexico City. | Bulletin of the World Health Organization | No Shigella-specific outcomes |
| Platts-Mills 2013 | Association between enteropathogens, diarrhea and growth in the mal-ed cohort | Am. J. Trop. Med. Hyg. | No Shigella-specific outcomes |
| Platts-Mills 2014 | Pathogen-specific etiology and burden of community diarrhea in the first two years of life in developing countries: Results from the mal-ED multisite cohort study | Am. J. Trop. Med. Hyg. | No follow-up/cross-sectional |
| Platts-Mills 2015 | Association between enteropathogens to moderate-to-severe malnutrition in children aged 6-24 months in Dhaka, Bangladesh (mal-ed): A casecontrol study | Am. J. Trop. Med. Hyg. | No follow-up/cross-sectional |
| Platts-Mills 2015 | Pathogen-specific burdens of community diarrhoea in developing countries: a multisite birth cohort study (MAL-ED). | The Lancet. Global health | No follow-up/cross-sectional |
| Platts-Mills 2022 | Impact of Biannual Mass Azithromycin Treatment on Enteropathogen Carriage in Children <5 Years Old in Niger. | Clin Infect Dis | No Shigella-specific outcomes |
| Poocharoen 1986 | The relative importance of various enteropathogens as a cause of diarrhoea in hospitalized children in Chiang Mai, Thailand. | Journal of diarrhoeal diseases research | No follow-up/cross-sectional |
| Pop 2014 | Diarrhea in young children from low-income countries leads to large-scale alterations in intestinal microbiota composition. | Genome biology | No follow-up/cross-sectional |
| Prado Camacho 1989 | A comparison of furazolidone and ampicillin in the treatment of invasive diarrhea. | Scandinavian journal of gastroenterology. Supplement | No Shigella-specific outcomes |
| Praharaj 2019 | Diarrheal Etiology and Impact of Coinfections on Rotavirus Vaccine Efficacy Estimates in a Clinical Trial of a Monovalent Human-Bovine (116E) Oral Rotavirus Vaccine, Rotavac, India. | Clinical infectious diseases : an official publication of the Infectious Diseases Society of America | No follow-up/cross-sectional |
| Prentice-Mott 2020 | Detection of enteric pathogens and continuation of diarrhea among children with moderate-tosevere diarrhea enrolled in the vaccine impact on diarrhea in africa (Vida) study: Kenya, 2015-2018 | Am. J. Trop. Med. Hyg. | Fewer than 5 children with shigella |
| Rahman 1990 | Acute lower respiratory tract infections in hospitalized patients with diarrhea in Dhaka, Bangladesh. | Reviews of infectious diseases | No Shigella-specific outcomes |
| Rahman 1997 | Absorption of macronutrients and nitrogen balance in children with dysentery fed an amylase-treated energy-dense porridge. | Acta paediatrica (Oslo, Norway : 1992) | No Shigella-specific outcomes |
| Rahman 2001 | Simultaneous zinc and vitamin A supplementation in Bangladeshi children: randomised double blind controlled trial. | BMJ (Clinical research ed.) | Shigella not detected/No testing |
| Randremanana 2016 | Etiologies, Risk Factors and Impact of Severe Diarrhea in the Under-Fives in Moramanga and Antananarivo, Madagascar. | PloS one | No Shigella-specific outcomes |
| Ranjbar 2010 | Fatality due to shigellosis with special reference to molecular analysis of Shigella sonnei strains isolated from the fatal cases | Iran. J. Clin. Infect. Dis. | No outcomes reported among <5 yr except mortality |
| Rasolofo-Razanamparany 2001 | Predominance of serotype-specific mucosal antibody response in Shigella flexneri-infected humans living in an area of endemicity. | Infection and immunity | Outcomes not stratified among <5 yr |
| Rawashdeh 1994 | Shigellosis in Jordanian children: a clinico-epidemiologic prospective study and susceptibility to antibiotics. | Journal of tropical pediatrics | Outcomes not stratified among <5 yr |
| Ray 1985 | Recent dysentery and gastroenteritis outbreak in some areas of Hooghly Distt. (West Bengal). | The Journal of communicable diseases | No follow-up/cross-sectional |
| Raza 1995 | Lactobacillus GG promotes recovery from acute nonbloody diarrhea in Pakistan. | The Pediatric infectious disease journal | Shigella not detected/No testing |
| Raza 2016 | Shigella infections: A two year experience in cancer patients. | JPMA. The Journal of the Pakistan Medical Association | Outcomes not stratified among <5 yr |
| Riewpaiboon 2008 | Predicting treatment cost for bacterial diarrhoea at a regional hospital in Thailand. | Journal of health, population, and nutrition | Outcomes not stratified among <5 yr |
| Rivas 1996 | [Cases of gastroenteritis associated to Vibrio cholerae no 01 in Oran, Salta]. | Revista Argentina de microbiologia | Fewer than 5 children with shigella |
| Rogerie 1986 | Comparison of norfloxacin and nalidixic acid for treatment of dysentery caused by Shigella dysenteriae type 1 in adults. | Antimicrobial agents and chemotherapy | Doesn't include children <5 years |
| Rollins 1995 | Epidemic Shigella dysenteriae type 1 in Natal. | Journal of tropical pediatrics | No follow-up/cross-sectional |
| Roy 1992 | Impact of zinc supplementation on intestinal permeability in Bangladeshi children with acute diarrhoea and persistent diarrhoea syndrome. | Journal of pediatric gastroenterology and nutrition | No Shigella-specific outcomes |
| Saito-Benz 2012 | Observational study on effect of HIV infection on presentation and outcome of diarrhoeal diseases in children less than 5 years of age in kwazulu-natal, south africa | Arch. Dis. Child. | Shigella not detected/No testing |
| Salam 1998 | Randomised comparison of ciprofloxacin suspension and pivmecillinam for childhood shigellosis. | Lancet (London, England) | Outcomes not stratified among <5 yr |
| Samuel 2017 | Effectiveness of a program intervention with reduced-iron MNPS on morbidity, iron status and child growth in young children in Ethiopia | Ann. Nutr. Metab. | Shigella not detected/No testing |
| Santos Ocampo 1989 | Aeromonas-associated diarrhea in Filipino children. | Rivista europea per le scienze mediche e farmacologiche = European review for medical and pharmacological sciences = Revue europeenne pour les sciences medicales et pharmacologiques | Shigella not detected/No testing |
| Sarangi 2015 | Prevalence of rotaviral diarrhoea in under-five hospitalized children in a tertiary care hospital of Eastern India | Egypt. Pediatr. Assoc. Gaz. | No Shigella-specific outcomes |
| Sarker 2021 | Characteristics of Rotavirus, ETEC, and Vibrio Cholerae Among Under 2-year Children Attending an Urban Diarrheal Disease Hospital in Bangladesh. | J Prim Care Community Health | No follow-up/cross-sectional |
| Sazawal 1996 | Zinc supplementation reduces the incidence of persistent diarrhea and dysentery among low socioeconomic children in India. | The Journal of nutrition | Shigella not detected/No testing |
| Scaletsky 1999 | [Association of patterns of Escherichia coli adherence to HEp-2 cells with acute and persistent diarrhea]. | Arquivos de gastroenterologia | Fewer than 5 children with shigella |
| Schiaffino 2022 | Antibiotic Use and Stewardship Practices in a Pediatric Community-Based Cohort Study in Peru: Shorter Would be Sweeter. | Clin Infect Dis | Shigella not detected/No testing |
| Schilling 2010 | Characteristics and etiology of moderate-tosevere diarrhea of prolonged or persistent duration among children less than five years old in rural western Kenya, 2008-2009 | Am. J. Trop. Med. Hyg. | No follow-up/cross-sectional |
| Schnee 2017 | Etiology-specific diarrhea by qPCR and linear growth deficits in Bangladeshi infants | Am. J. Trop Med. Hyg | Duplicate data |
| Sengupta 1990 | Multidrug resistant epidemic shigellosis in a village in west Bengal, 1984. | Indian journal of public health | No follow-up/cross-sectional |
| Sethabutr 1994 | Detection of Shigella and enteroinvasive Escherichia coli by PCR in the stools of patients with dysentery in Thailand. | Journal of diarrhoeal diseases research | No Shigella-specific outcomes |
| Shamsizadeh 2012 | Neurological manifestations of shigellosis in children in southwestern Iran. | Pediatrics international : official journal of the Japan Pediatric Society | Outcomes not stratified among <5 yr |
| Sharif 2017 | The Role of Probiotics in the Treatment of Dysentery: a Randomized Double-Blind Clinical Trial. | Probiotics and antimicrobial proteins | Shigella not detected/No testing |
| Shen 2019 | Dynamic construction of gut microbiota may influence allergic diseases of infants in Southwest China. | BMC microbiology | Fewer than 5 children with shigella |
| Shimelis 2004 | Clinical profile of acute renal failure in children admitted to the department of pediatrics, Tikur Anbessa Hospital. | Ethiopian medical journal | No follow-up/cross-sectional |
| Shimelis 2020 | Aetiology of acute febrile illness among children attending a tertiary hospital in southern Ethiopia. | BMC infectious diseases | No follow-up/cross-sectional |
| Shkarin 1983 | [Epidemiology of bacillary dysentery in Algeria. I. The epidemiological aspects of dysentery in Algeria]. | Zhurnal mikrobiologii, epidemiologii i immunobiologii | Outcomes not stratified among <5 yr |
| Shwe 2002 | Blood culture isolates from children admitted to Medical Unit III, Yangon Children's Hospital, 1998. | The Southeast Asian journal of tropical medicine and public health | No follow-up/cross-sectional |
| Simpore 2009 | Aetiology of acute gastro-enteritis in children at Saint Camille Medical Centre, Ouagadougou, Burkina Faso. | Pakistan journal of biological sciences : PJBS | No follow-up/cross-sectional |
| Sinha 2016 | Circulating Gut-Homing (Œ±4Œ≤7+) Plasmablast Responses against Shigella Surface Protein Antigens among Hospitalized Patients with Diarrhea. | Clinical and vaccine immunology : CVI | Doesn't include children <5 years |
| Sircar 1984 | A longitudinal study of diarrhoea among children in Calcutta communities | Indian J Med Res | No Shigella-specific outcomes |
| Sirivichayakul 1998 | Severe shigellosis in childhood. | The Southeast Asian journal of tropical medicine and public health | No follow-up/cross-sectional |
| Sowmyanarayanan 2009 | Nitric oxide production in acute gastroenteritis in Indian children. | Transactions of the Royal Society of Tropical Medicine and Hygiene | No Shigella-specific outcomes |
| Sreenivasan 2012 | Sequelae of moderate-to-severe diarrhea among young children in Western Kenya, 2008-2011 | Am. J. Trop. Med. Hyg. | No Shigella-specific outcomes |
| Srivastava 1990 | Acute renal failure in north Indian children. | The Indian journal of medical research | No Shigella-specific outcomes |
| Srivastava 1991 | Hemolytic uremic syndrome in children in northern India. | Pediatric nephrology (Berlin, Germany) | No Shigella-specific outcomes |
| Stanton 1989 | Parasitic, bacterial and viral pathogens isolated from diarrhoeal and routine stool specimens of urban Bangladeshi children | J Trop Med Hyg | No follow-up/cross-sectional |
| Stoll 1982 | Epidemiologic and clinical features of patients infected with Shigella who attended a diarrheal disease hospital in Bangladesh. | The Journal of infectious diseases | No outcomes reported among <5 yr except mortality |
| Sutmoller 1982 | An outbreak of gastroenteritis caused by both rotavirus and Shigella sonnei in a private school in Rio de Janeiro. | The Journal of hygiene | No follow-up/cross-sectional |
| Sutra 1990 | The pattern of diarrhea in children in Khon Kaen, northeastern Thailand: I. The incidence and seasonal variation of diarrhea. | The Southeast Asian journal of tropical medicine and public health | Shigella not detected/No testing |
| Sutra 2012 | Burden of acute, persistent and chronic diarrhea, Thailand, 2010. | Journal of the Medical Association of Thailand = Chotmaihet thangphaet | Shigella not detected/No testing |
| Talbert 2019 | Mortality after inpatient treatment for diarrhea in children: a cohort study. | BMC medicine | Shigella not detected/No testing |
| Tall 1993 | [Diarrheic diseases in children. Rationalization of fecal test]. | Bulletin de la Societe de pathologie exotique (1990) | Shigella not detected/No testing |
| Taniuchi 2012 | A prospective study on potential causes of diarrhea in bangladeshi children in the first year of life using a pcr-luminex based detection of 28 most common enteropathogens | Am. J. Trop. Med. Hyg. | No Shigella-specific outcomes |
| Taylor 1989 | Introduction and spread of multi-resistant Shigella dysenteriae I in Thailand. | The American journal of tropical medicine and hygiene | No outcomes reported among <5 yr except mortality |
| Taylor 1993 | Campylobacter immunity and quantitative excretion rates in Thai children. | The Journal of infectious diseases | No Shigella-specific outcomes |
| Teka 1996 | Risk factors for deaths in under-age-five children attending a diarrhoea treatment centre. | Acta paediatrica (Oslo, Norway : 1992) | No Shigella-specific outcomes |
| Thanh 1992 | Clinical aspects of acute vs persistent diarrhea in Ho Chi Minh City, Vietnam. | Acta paediatrica (Oslo, Norway : 1992). Supplement | Shigella not detected/No testing |
| Thapa 1995 | Shigellosis in children from north India: a clinicopathological study. | Journal of tropical pediatrics | Outcomes not stratified among <5 yr |
| Thisyakorn 1992 | Shigellosis in Thai children: epidemiologic, clinical and laboratory features. | The Pediatric infectious disease journal | Outcomes not stratified among <5 yr |
| Thompson 2016 | Clinical implications of reduced susceptibility to fluoroquinolones in paediatric Shigella sonnei and Shigella flexneri infections. | The Journal of antimicrobial chemotherapy | Outcomes not stratified among <5 yr |
| Tickell 2020 | The effect of acute malnutrition on enteric pathogens, moderate-to-severe diarrhoea, and associated mortality in the Global Enteric Multicenter Study cohort: a post-hoc analysis. | The Lancet. Global health | No outcomes reported among <5 yr except mortality |
| Torres 2000 | Association of diarrhoea and upper respiratory infections with weight and height gains in Bangladeshi children aged 5 to 11 years. | Bulletin of the World Health Organization | Shigella not detected/No testing |
| Trabulsi 1988 | Diarrheal disease in children in S√£o Paulo. | Kansenshogaku zasshi. The Journal of the Japanese Association for Infectious Diseases | No follow-up/cross-sectional |
| Umoh 1983 | Epidemiological features of an outbreak of gastroenteritis/cholera in Katsina, Northern Nigeria. | The Journal of hygiene | No Shigella-specific outcomes |
| Uysal 1997 | Campylobacter jejuni gastroenteritis in Turkish children. | Infection | No follow-up/cross-sectional |
| Vaktskjold 2010 | Infant growth disparity in the Khanh Hoa province in Vietnam: a follow-up study. | BMC pediatrics | Shigella not detected/No testing |
| Vergara 1992 | [Identification of enteropathogens in infantile diarrhea in a study performed in the city of Posadas, Misiones, Rep√∫blica Argentina]. | Revista latinoamericana de microbiologia | No follow-up/cross-sectional |
| Victora 1992 | Deaths due to dysentery, acute and persistent diarrhoea among Brazilian infants. | Acta paediatrica (Oslo, Norway : 1992). Supplement | Shigella not detected/No testing |
| Victora 1993 | International differences in clinical patterns of diarrhoeal deaths: a comparison of children from Brazil, Senegal, Bangladesh, and India. | Journal of diarrhoeal diseases research | Shigella not detected/No testing |
| Vinh 2011 | A multi-center randomized trial to assess the efficacy of gatifloxacin versus ciprofloxacin for the treatment of shigellosis in Vietnamese children. | PLoS neglected tropical diseases | Outcomes not stratified among <5 yr |
| Vubil 2018 | Antibiotic resistance and molecular characterization of shigella isolates recovered from children aged less than 5 years in Manhi√ßa, Southern Mozambique. | International journal of antimicrobial agents | No outcomes reported among <5 yr except mortality |
| Wahed 1999 | Retinol concentrations in liver and serum among children who died in a diarrheal hospital in Bangladesh | Nutr. Res. | No Shigella-specific outcomes |
| Walker 2014 | Pathogen-specific mortality among infants and young children with moderate-to-severe diarrhea-Western Kenya, 2008-2011 | Am. J. Trop. Med. Hyg. | No outcomes reported among <5 yr except mortality |
| Xu 2009 | [A multicentre study of Shigella spp in Henan province, during 2000-2007]. | Zhonghua liu xing bing xue za zhi = Zhonghua liuxingbingxue zazhi | No follow-up/cross-sectional |
| Ye 1986 | [A study on etiology and epidemiology of 1446 patients with diarrhea]. | Zhonghua yu fang yi xue za zhi [Chinese journal of preventive medicine] | No follow-up/cross-sectional |
| Yousefi 2018 | A food poisoning outbreak by Shigella boydii in Kerman-Iran | Arch. Clin. Infect. Dis. | Doesn't include children <5 years |
| Yunus 1982 | Clinical trial of ampicillin v. trimethoprim-sulphamethoxazole in the treatment of Shigella dysentery. | The Journal of tropical medicine and hygiene | Outcomes not stratified among <5 yr |
| Zaki 1986 | The detection of enteropathogens in acute diarrhea in a family cohort population in rural Egypt. | The American journal of tropical medicine and hygiene | No follow-up/cross-sectional |
| Zaman 1991 | Surveillance of shigellosis in rural Bangladesh: a 10 years review. | JPMA. The Journal of the Pakistan Medical Association | No follow-up/cross-sectional |
| Zimmerman 1989 | Acute bacillary dysentery in Nepal: A retrospective view | J. INST. MED. | Full text not available |
|  | Shigella dysenteriae type 1‚ÄìGuatemala, 1991. | MMWR. Morbidity and mortality weekly report | No follow-up/cross-sectional |
|  | [Epidemiologic survey of an outbreak of food-borne bacillary dysentery]. | Zhonghua liu xing bing xue za zhi = Zhonghua liuxingbingxue zazhi | No outcomes reported among <5 yr except mortality |
|  | [Analysis of the effects of compound "Xiaolijun" in the prevention of chronic bacillary dysentery]. | Zhonghua yi xue za zhi | No Shigella-specific outcomes |
|  | Multicenter, randomized, double blind clinical trial of short course versus standard course oral ciprofloxacin for Shigella dysenteriae type 1 dysentery in children. | The Pediatric infectious disease journal | Outcomes not stratified among <5 yr |
|  | The topic of this month. Shigellosis, Japan 1999-2000 | Jpn. J. Infect. Dis. | Study not in LMIC |

# **Appendix 4. Included studies where the number of children with *Shigella* was not specified**

| **Study** | **Total number of participants < 5years** | **# of stools tested** | **# of stools positive for shigella** | **# of diarrhea stools positive** | **# non-diarrhea stools positive** | **# *Shigella*-attributed diarrhea episodes** | **Estimated # of children with *Shigella*** | **Notes** |
| --- | --- | --- | --- | --- | --- | --- | --- | --- |
| Baqui et al, 1988 | 705 | - | 23 | 20 | 3 | - | at least 20 | 10 positive stools at days 1-3 of diarrhea episode, and 10 different children positive at days 15-17; plus 3 routine samples positive |
| Black et al, 1982 | 197 | - | - | 118 | 20 | - | 118 diarrhea episodes | 118 diarrhea episodes and 20 healthy specimens |
| Black et al, 1984 | 197 | - | 56 | - | - | - | at least 60 diarrhea episodes | A total of 197 children participating in the study, and shigella incidence and days of follow-up are reported by different strata, so it's possible to back-calculate the number of shigella diarrhea episodes. |
| Donowitz et al, 2021 | 250 | - | Not reported; >5% prevalence | - | - | - | at least 8 | "Only pathogens with at least a 5% prevalence across all samples were included in the analysis." Multivariable regressions were conducted on 179 and 162 participants, respectively. Thus, we can conclude that at least 8 participants in this study had *shigella*. |
| Huttly et al, 1989 | NS | - | - | Not reported, 4286 diarrhea episodes | 221 | - | at least 221 diarrhea episodes | Not reported, 221 episodes with shigella confirmed |
| Luoma et al, 2022 | 604 | 587 | 60 |  |  |  | 60 | 10.2% of samples (or children-not clear) were positive for *Shigella*. |
| Nasrin et al, 2021 | 7545 |  | 1001 |  |  |  | >40 | 1001 positive stools (606 among 0-23 month-olds (Table 2)) |
| Perin et al, 2020 | 68 | - | - | - | - | - | at least 12 | A total of 68 children were included in the study; figure 3 shows the abundance of 40 most common genera in stool samples at baseline and 18-month follow-up among Mirzapur, Bangladesh, age-matched pairs, for the height/length for age growth criteria. Pairs were constructed so that those in the tertile of highest growth were matched to a child in the lowest growth tertile whose age at baseline was within one month. Counting only those at 18 months in the diagram, there are at least 12 children in the study who have Esherichica/Shigella. |
| Rogawski et al, 2018 | 1469 | 35,622 | - | - | - | - | at least 119 | 1469 total participants in the study, with 1202 with anthropometry data at age 5 years. From Table 1, we can calculate the mean stools per child ranging from 18-27 (add diarrhea and non-diarrhea stools together and divide by n children). 3,237 non-diarrheal stools. If we assume that there were 18 stools per child at the very least, then we can say there were at least 179 children. If we assume that each child gave up to 27 stool samples, we can assume 119 children at least who had shigella. |
| Rogawski McQuade et al, 2020 | 1715 | - | 4744 | 1239 | 3505 | 755 | at least 71 | Out of 41405 stools total, there was shigella detection in 4744 (or 11.5%) and 1715 children included in the study. If we assume that each child gave 24 stools (41405/1715) then we can say that there were at least 71 children with shigella |
| Rogawski McQuade et al., 2022 | 451 | ~9922 | ~793 | - | - | - | at least 36 | Median of 22 stools per child; 8% of stools positive for shigella; 17% in Tanzania; and 5% in Brazil and South Africa. |
| Schnee et al, 2018 | 700 | 1993 | - | - | - | 125 | 125 diarrhea episodes | 125 diarrhea episodes attributable to shigella |

# **Appendix 5. Quality scores of included studies**

| **Study** | **STROBE Methods Quality Score** |
| --- | --- |
| Abu-Elyazeed 2004 | 10 |
| Ahmed 2001 | 10 |
| Anders 2015 | 9 |
| Andersson 2017 | 8 |
| Ballard 2022 | 8 |
| Baqui 1992 | 6 |
| Black 1984 | 10 |
| Black 1982 | 9 |
| Black 1984 | 8 |
| Butler 1984 | 7 |
| Cravioto 1990 | 6 |
| Das 2015 | 9 |
| Das 2021 | 10 |
| Donowitz 2021 | 9 |
| Dutta 1991 | 5 |
| Dutta 1992 | 8 |
| Echeverria 1988 | 4 |
| Fujita 1990 | 6 |
| Gaensbauer 2019 | 9 |
| George 2018 | 8 |
| Guh 2008 | 9 |
| Henry 1992 | 9 |
| Househam 1990 | 5 |
| Huskins 1994 | 9 |
| Huttly 1989 | 9 |
| Kabir 1993 | 7 |
| Kabir 1998 | 7 |
| Khan 1984 | 5 |
| Luoma 2022 | 8 |
| Mazumder 1996 | 6 |
| Mazumder 1997 | 8 |
| Mitra 1998 | 7 |
| Nasrin 2021 | 10 |
| Ndungo 2022 | 8 |
| Perin 2020 | 9 |
| Platts-Mills 2014 | 9 |
| Platts-Mills 2017 | 10 |
| Platts-Mills 2021 | 9 |
| Rahman 1995 | 7 |
| Ramiro Cruz 1994 | 6 |
| Rampengan 1982 | 6 |
| Raqib 2004 | 7 |
| Riewpaiboon 2008 | 8 |
| Rodriguez 1989 | 10 |
| Rogawski 2018 | 9 |
| Rogawski McQuade 2020 | 9 |
| Rogawski McQuade 2022 | 9 |
| Roy 2008 | 9 |
| Schnee 2018 | 10 |
| Taylor 1987 | 9 |
| Versloot 2018 | 7 |
| Zimmermann 2019 | 10 |

Abbreviations: STROBE, Strengthening the Reporting of Observational Studies in Epidemiology

# **Appendix 6. Summary of other anthropometric measures**

| **Outcome** | **Follow-up time frame** | **Paper** | **# with *Shigella*** | **Comparison group** | **Effect measure** |
| --- | --- | --- | --- | --- | --- |
| **Mean change/difference in weight-for-height Z-score (WHZ)** | | | | |  |
|  | 21 days | Kabir 1993 | 69 | Day 1 to Day 21 among children fed high-protein diet | 0.89 (SD: 0.40) |
|  | 21 days | Kabir 1993 | 69 | Day 1 to Day 21 among children fed a standard-protein diet (of comparable total energy) | 0.64 (SD: 0.46) |
|  | 6 months | Kabir 1998 | 59 | Day 1 to 6 months among children fed high-protein diet | 0.26 (SD: 0.49) |
|  | 6 months | Kabir 1998 | 59 | Day 1 to 6 months among children fed a standard-protein diet (of comparable total energy) | 0.35 (SD: 0.53) |
|  | ~60 days | Das 2021 | 591 | Comparing children with *Shigella* detected 60 days prior to those without *Shigella* detected, adjusted for confounders, co-infections | -0.11 (95% CI: -0.21, -0.001) |
|  | 2 years | Rogawski 2018 | NS; 1469 participants | Comparing children with high (90th percentile) to low (10th percentile) *Shigella* prevalence in non-diarrheal stools, adjusted for site, enrolment WAZ and LAZ, sex, SES, exclusive breastfeeding in the first 6 months of life, and maternal height | -0.07 (95% CI: -0.22, 0.08). |
| **Odds of wasting (WHZ <-2)** | | | |  |  |
|  | 9 months | George 2018 | 71 | Odds of wasting at 9-month follow-up comparing children with *Shigella* detected at baseline to those without *Shigella* detected | 0.81 (95% CI 0.22, 2.93) |
| **Mean WHZ at discharge** | |  |  |  |  |
|  | At hospital discharge | Mitra 1998 | 66 | Mean WHZ at discharge among children hospitalized with *S. dysenteriae* | -1.7 (SD 1.1) |
|  | At hospital discharge | Mitra 1998 | 66 | Mean WHZ at discharge among children hospitalized with *Shigella* species other than dysenteriae | -1.3 (SD: 0.8) |
| **Mean change/difference in weight-for-height (kg/m)** | | | |  |  |
|  | 10 days | Mazumder 1997 | 75 | Day 0 to Day 10 in the group receiving 10 days of an energy-dense milk formula | 6.71 (95% CI: 5.30, 98.13) |
|  | 10 days | Mazumder 1997 | 75 | Day 0 to Day 10 in the control group | 2.95 (95% CI: 1.78, 4.12) |
|  | 40 days | Mazumder 1997 | 75 | Day 0 to Day 40 in the group receiving 10 days of an energy-dense milk formula | 8.15 (95% CI: 6.14, 10.20) |
|  | 40 days | Mazumder 1997 | 75 | Day 0 to Day 40 in the control group | 4.81 (95% CI: 3.20, 6.43) |
| **Mean change in weight-for-age Z-score (WAZ)** | | | |  |  |
|  | 21 days | Kabir 1993 | 69 | Day 1 to Day 21 among children fed high-protein diet | 0.75 (SD: 0.27) |
|  | 21 days | Kabir 1993 | 69 | Day 1 to Day 21 among children fed a standard-protein diet (of comparable total energy) | 0.52 (SD: 0.38) |
|  | 6 months | Kabir 1998 | 59 | Day 1 to 6 months among children fed high-protein diet | 0.45 (SD: 0.34) |
|  | 6 months | Kabir 1998 | 59 | Day 1 to 6 months among children fed a standard-protein diet (of comparable total energy) | 0.38 (SD: 0.57) |
|  | ~60 days | Das 2021 | 591 | Comparing children with *Shigella* detected 60 days prior to those without *Shigella* detected, adjusted for confounders, co-infections | -0.06 (95% CI: -0.17, 0.05) |
|  | 5 months | Platts-Mills 2017 | 93 | Among children with malnutrition, the change in WAZ following a nutritional intervention among those with *Shigella* detected at baseline, compared to those without *Shigella* detected | 0.04 (95% CI: -0.9, 0.17) |
|  | 2 years | Rogawski 2018 | NS; 1469 participants | Comparing children with high (90th percentile) to low (10th percentile) *Shigella* prevalence in non-diarrheal stools, adjusted for site, enrolment WAZ and LAZ, sex, SES, exclusive breastfeeding in the first 6 months of life, and maternal height | -0.12 (95% CI: -0.26, 0.02) |
| **Odds of underweight (WAZ <-2)** | | | |  |  |
|  | 9 months | George 2018 | 71 | Odds of underweight at 9-month follow-up comparing children with *Shigella* detected at baseline to those without *Shigella* detected | 1.69 (95% CI: 0.82, 3.49) |
| **Mean WAZ at discharge** | |  |  |  |  |
|  | At hospital discharge | Mitra 1998 | 66 | WAZ at discharge among children hospitalized with *S. dysenteriae* | -2.5 (SD:1.3) |
|  | At hospital discharge | Mitra 1998 | 66 | WAZ at discharge among children hospitalized with *Shigella* species other than dysenteriae | -2.0 (SD: 0.7) |
| **Mean change in weight-for-age (kg/m)** | | | | |  |
|  | 10 days | Mazumder 1997 | 75 | Day 0 to Day 10 in the group receiving 10 days of an energy-dense milk formula | 6.29 (95% CI: 5.0, 7.59) |
|  | 10 days | Mazumder 1997 | 75 | Day 0 to Day 10 in the control group | 2.79 (95% CI: 1.79, 3.80) |
|  | 40 days | Mazumder 1997 | 75 | Day 0 to Day 40 in the group receiving 10 days of an energy-dense milk formula | 8.21 (95% CI: 6.52, 9.91) |
|  | 40 days | Mazumder 1997 | 75 | Day 0 to Day 40 in the control group | 5.10 (95% CI: 3.72, 6.48) |

Abbreviations: CI, confidence interval; SD, standard deviation; WHZ, weight-for height z-score; WLZ, weight-for-length z-score

# **Appendix 7. Summary of additional outcomes**

| **Outcome** | **Follow-up time frame** | **Paper** | **# with *Shigella*** | **Measurement/ comparison group** | **Effect measure** |
| --- | --- | --- | --- | --- | --- |
| **Neurodevelopmental outcomes** | | |  |  |  |
|  | 24 months | Donowitz 2021 | NS | Change in cognitive score^a^ per 1 episode of diarrhea attributable to *Shigella* | 0.21 (-0.58, 1.01); p=0.60 |
|  | 24 months | Donowitz 2021 | NS | Change in language (receptive and expressive) score^a^ per 1 episode of diarrhea attributable to *Shigella* | -0.74 (-1.68, 0.21); p=0.13 |
|  | 24 months | Donowitz 2021 | NS | Change in motor skills (fine and gross) score^a^ per 1 episode of diarrhea attributable to *Shigella* | 0.57 (-0.33, 1.48); p =0.22 |
|  | 6-8 years | Rogawski McQuade 2022 | NS; 451* | z-score difference in reasoning skills score comparing high vs. low *Shigella* prevalence over 24-months | -0.12 (-0.38, 0.13) |
|  | 6-8 years | Rogawski McQuade 2022 | NS; 451* | z-score difference in reasoning skills score per 1 log increase in *Shigella* over 24-months | -0.12 (-0.34, 0.09) |
|  | 6-8 years | Rogawski McQuade 2022 | NS; 451* | z-score difference in phonemic fluency score comparing high vs. low *Shigella* prevalence over 24-months | -0.12 (-0.36, 0.13) |
|  | 6-8 years | Rogawski McQuade 2022 | NS; 451* | z-score difference in phonemic fluency score per 1 log increase in *Shigella* over 24-months | -0.14 (-0.35, 0.07) |
|  | 6-8 years | Rogawski McQuade 2022 | NS; 451* | z-score difference in semantic fluency score comparing high vs. low *Shigella* prevalence over 24-months | -0.13 (-0.40, 0.13) |
|  | 6-8 years | Rogawski McQuade 2022 | NS; 451* | z-score difference in semantic fluency score per 1 log increase in *Shigella* over 24-months | -0.16 (-0.38, 0.07) |
| **Gut markers of inflammation/ Environmental enteric dysfunction (EED)** | | |  |  |  |
|  | 30 days | Raqib 2004 | 56 | Geometric mean (standard error of mean) of myeloperoxidase (U/mg total protein) at different days of follow up in zinc (intervention) and control group | Zinc group: day 1: 3.2 (4); day 7: 0.12 (1.8); day 30: 0.18 (0.7)  Control group: day 1: 3 (3); day 7: 0.4 (1.3); day 30: 0.65 (1)  No significant differences between groups or within groups |
|  | 30 days | Raqib 2004 | 56 | Geometric mean (standard error of mean) of superoxidase (U/mg total protein) at different days of follow up in Zinc (intervention) and control group | Zinc group: day 1: 0.2 (0.06); day 7: 0.34 (0.6); day 30: 0.15 (0.07)  Control group: day 1: 0.3 (0.2); day 7: 0.2 (0.3); day 30: 0.2 (0.1)  No significant differences between groups or within groups |
|  | 30 days | Raqib 2004 | 56 | Geometric mean (standard error of mean) of nitrate (umol/umol creatinine) at different days of follow up in Zinc (intervention) and control group | Zinc group: day 1: 1.2 (0.3); day 7: 1 (0.2); day 30: 0.8 (0.1)  Control group: day 1: 0.8 (0.2); day 7: 0.73 (0.1); day 30: 0.72 (0.09)  No significant differences between groups or within groups |
|  | 30 days | Raqib 2004 | 56 | Geometric mean (standard error of mean) of IL-1B (pg/mL) in stool extracts at different days of follow up in Zinc (intervention) and control group | Zinc group: day 1: 2,562 (133); day 7: 38 (22); day 30: 92 (16)  Control group: day 1: 2,577 (871); day 7: 56 (112); day 30: 68 (19)  Significant difference over time (p = 0.001); No significant difference between groups |
|  | 30 days | Raqib 2004 | 56 | Geometric mean (standard error of mean) of IL-2 (pg/mL) in stool extracts at different days of follow up in Zinc (intervention) and control group | Zinc group: day 1: 72 (10); day 7: 79 (12); day 30: 70 (12)  Control group: day 1: 79 (12); day 7: 78 (3); day 30: 76 (2)  No significant differences between groups or within groups |
|  | 30 days | Raqib 2004 | 56 | Geometric mean (standard error of mean) of IFN-gamma (pg/mL) in stool extracts at different days of follow up in Zinc (intervention) and control group | Zinc group: day 1: 12 (2); day 7: 13 (1); day 30: 13 (3)  Control group: day 1: 12 (2); day 7: 15 (2.3); day 30: 14 (1.3)  No significant differences between groups or within groups |
| **Systemic markers of inflammation** | | |  |  |  |
|  | 30 days | Raqib 2004 | 56 | Mean Interleukin-2 and interferon-gamma response in peripheral blood mononuclear cells to mitogen (PHA) stimulation over time (Days 1, 7, and 30) | No significant difference in zinc treatment or control group or within each group over time. |
|  | 30 days | Raqib 2004 | 56 | Mean serum C-Reactive protein (CRP) in mg/dL (SD) in control group on Day 1, 7, and 30 | Day 1: 9 (0.3); Day 7: 0.5 (0.08); Day 30: 0.5 (0.07) p=0.001 (No difference compared to zinc group.) |
|  | 2 years | Schnee 2018 | 125 | Increase in serum C-reactive protein levels (no units specified) per diarrhea episode attributable to *Shigella*, over the entire interval of measurement | 0.24 (95% CI: 0.03, 0.49) |
|  | 2 years | Schnee 2018 | 125 | Increase in serum C-reactive protein levels per diarrhea episode attributable to *Shigella* in the 4-week interval prior to CRP measurement. | 0.24 (95% CI: -0.13, 0.79) |
| **Antigen-specific antibody response** | | | |  |  |
|  | 21-28 days | Echeverria 1988 | 19 | % children who developed antibodies to plasmid-encoded antigens (by immunoblot) | 42% (8/19) |
|  | 6-24 hours | Echeverria 1988 | 19 | % children who developed >= 4-fold rise (measured in acute serum) in IgG antibodies to water-extracted antigens of M90T. | 42% (8/19) overall; < 2 years: 2/9; 2-4 years: 6/10 |
|  | 6-24 hours | Echeverria 1988 | 19 | Geometric mean (range) titers of acute sera to water-extracted antigens of M90T for IgG, IgM, and IgA | IgG: 1659 (200-25600); IgM: 480 (500-3200); IgA: 60 (50-400) |
|  | 30 days | Raqib 2004 | 56 | Geometric mean (standard error of the mean) of Ipa-specific IgG response at different days of follow up in Zinc (intervention) and control group | Zinc group: day 1: 80 (9); day 7: 117 (10); day 30: 204 (34)  Control group: day 1: 83 (10); day 7: 97 (11); day 30: 161 (18)  Significant difference over time (p < 0.001); No significant difference between groups |
|  | 30 days | Raqib 2004 | 56 | Geometric mean (standard error of the mean) for antigen-specific antibody titers: (Plasma): LPS-IgA, LPS-IgG, Ipa-IgA, Ipa-IgG; (Stool): s-IgA, LPS-IgA, Ipa-IgA at 1,7, and 30 days of follow up in Zinc (intervention) and control group | Only plasma LPS-specific IgA (p = 0.012) and stool s-IgA (p = 0.026) had a statistically significant association with time (combining both groups) |
| **Microbiome composition** | | |  |  |  |
|  | 2 years | Ndungo 2022 | 30 | Alpha diversity as measured by Shannon Diversity Index, comparing *Shigella* cases to matched controls | Cases: 2.90; Controls: 3.16; p=0.52 |
|  | 2 years | Ndungo 2022 | 30 | Alpha diversity as measured by Shannon Diversity Index, comparing symptomatic vs asymptomatic *Shigella* infection | Symptomatic: 3.24; Asymptomatic: 3.41; p=0.65 |
|  | 2 years | Ndungo 2022 | 30 | Alpha diversity as measured by Shannon Diversity Index, comparing *Shigella* attributed diarrhea vs. other-cause diarrhea | *Shigella*: 3.07; Other-cause: 3.04; p=0.95 |
|  | 2 years | Ndungo 2022 | 30 | Mean relative abundance of 10 most abundant taxa following diarrhea episode comparing *Shigella* cases to controls. | *Fusicatenibacter saccharivorans, Lachnospiraceae* NK4A136, Facklamia hominis, and Holdemanella biformisgroup significantly more abundant in cases than controls |
| **Leukemoid reaction** | |  |  |  |  |
|  | Duration of hospitalization | Butler 1984 | 2,172 | % shigellosis cases with leukemoid reaction (one or more white blood cell counts of 50,000/cu mm or greater) | 4.6% (99/2,172) |
| **Stool pH/ water content** | |  |  |  |  |
|  | 1.1-2.7 months | Fujita 1990 | 5 | Mean water content in diarrheal specimens vs. recovery specimens | 95% vs. 74.8% |
|  | 1.1-2.7 months | Fujita 1990 | 5 | Mean pH of diarrheal stools vs. recovery stools | 6.52 vs. 5.99 |
| **Pathogen clearance** | |  |  | **Percent of *Shigella* cases that were no longer shedding at follow-up** | **% (fraction)** |
|  | 6 days | Rodriguez 1989 | 20 | without treatment (control) | 75% (3/4) |
|  | 6 days | Rodriguez 1989 | 20 | with furazolidone treatment | 40% (4/10) |
|  | 6 days | Rodriguez 1989 | 20 | with TMP-SMX treatment | 100% (6/6) |
|  | 14 days | Andersson 2017 | 42 | All cases | 50% (21/42) |
|  | 14 days | Andersson 2017 | 42 | with any antibiotic | 48% (16/33) |
|  | 14 days | Andersson 2017 | 42 | with no antibiotic | 56% (5/9) |
|  | 14 days | Andersson 2017 | 42 | with moderate wasting (weight-for-height Z-score <-2) | 36% (4/12) |
|  | 14 days | Andersson 2017 | 42 | without moderate wasting (weight-for-height Z-score >-2) | 55% (17/31) |
|  | 31 days | Gaensbauer 2019 | 112 | All cases | 21% (23/108) |
|  | At clinical stabilization (variable) | Versloot 2018 | 19 | with complicated severe acute malnutrition | 79% (15/19) |
| **Duration of *Shigella* excretion** | | |  |  |  |
|  | 10-12 days | Khan 1984 | 132 | Mean (range) duration of excretion of *Shigella* | 4.1 days (1-12) |
|  | 15-17 days | Baqui 1991 | 10 | Among cases of persistent diarrhea (14 or more days) with *Shigella* isolated at 1-3 days, the percent that were also positive for *Shigella* at 15-17 days | 0% (0/10) |
| **Repeat *Shigella* infections** | | |  |  |  |
| *Proportion of Shigella cases with subsequent Shigella infection* | | | | |  |
|  | 12 months | Anders 2015 | 108 | Proportion with a 2nd infection | 8% (9/108) |
|  | 12 months | Craviato 1990 | 11 | Proportion with a 2nd colonization (both asymptomatic) | 9% (1/11) |
|  | 24 months | Ramiro Cruz 1994 | 126 | Proportion with a 2nd infection | 35% (44/126) |
|  | 24 months | Ramiro Cruz 1994 | 126 | Proportion with a 2nd infection of the same serotype | 16% (20/126) |
|  | 24 months | Ramiro Cruz 1994 | 126 | Proportion with 3 infections | 8% (10/126) |
|  | 24 months | Ramiro Cruz 1994 | 126 | Proportion with 4 infections | 2% (3/126) |
|  | 24 months | Ramiro Cruz 1994 | 126 | Proportion with 5 infections | 2% (3/126) |
|  | 36 months | Abu-Elyazeed 2004 | 101 | Proportion with a 2nd infection | 20% (20/101) |
|  | 36 months | Abu-Elyazeed 2004 | 101 | Proportion with 3 infections | 5% (5/101) |
|  | 36 months | Abu-Elyazeed 2004 | 101 | Proportion with 4 infections | 1% (1/101) |
| *Interval between subsequent Shigella infections* | | | | |  |
|  | 12 months | Anders 2015 | 108 | Median (IQR) number of days between repeat infections | 73.8 days (23.6-97.1) |
|  | 12 months | Anders 2015 | 108 | 5th and 95th percentile for number of days between repeat infections | 8.7 days; 160.0 days |
|  | 24 months | Ramiro Cruz 1994 | 20 | Range of number of months between infections of the same serotype | 1-9 months |
| **Subsequent illness/infection** | | |  |  |  |
| *Subsequent Shigella infections of homologous serotype* | | | |  |  |
|  | 24 months | Ramiro Cruz 1994 | 20 | Proportion of new subsequent *Shigella* infections of homologous serotype that were asymptomatic (compared to the proportion of initial *Shigella* infections that were asymptomatic) | 65% (13/20) compared to 30% (6/20) of initial infections |
|  | 24 months | Ramiro Cruz 1994 | 20 | Proportion of new subsequent *Shigella* infections with diarrhea (note: 6/7 were co-infected with other diarrheal pathogens) | 35% (7/20) compared 45% (9/20) of initial infections |
|  | 24 months | Ramiro Cruz 1994 | 20 | Proportion of new subsequent *Shigella* infections with dysentery | 0% (0/20) compared to 25% (5/20) of initial infections |
| *Respiratory disease* | |  |  |  |  |
|  | 6 months | Roy 2008 | 56 | Mean # of respiratory infections (control group) | 3.3 (95% CI: 2.4, 4.6) |
|  | 6 months | Roy 2008 | 56 | Mean # of respiratory infections (zinc group) | 3.6 (95% CI: 2.5, 5.3) |
|  | 6 months | Kabir 1998 | 59 | Mean # of respiratory infections (high protein group) | 3.9 |
|  | 6 months | Kabir 1998 | 59 | Mean # of respiratory infections (standard protein group) | 4.3 |
| *Febrile Illness* | |  |  |  |  |
|  | 6 months | Roy 2008 | 56 | Mean # of febrile illnesses (control group) | 2.3 (95% CI: 1.7, 3.2). |
|  | 6 months | Roy 2008 | 56 | Mean # of febrile illnesses (zinc group) | 2.2 (95% CI: 1.6, 3.2) |
|  | 6 months | Kabir 1998 | 59 | Mean # of febrile illnesses (high protein group) | 2.5 |
|  | 6 months | Kabir 1998 | 59 | Mean # of febrile illnesses (standard protein group) | 2.8 |
| **Nutritional intake** | |  |  |  |  |
|  | 72 hours | Mazumder 1996 | 23 | Carbohydrate: Mean (and SEM) coefficient of percent nutrient absorption, compared between the control and test groups | Control: 66.9 (4.4); Test: 76.6 (4.8) |
|  | 72 hours | Mazumder 1996 | 23 | Fat: Mean (and SEM) coefficient of percent nutrient absorption, compared between the control and test groups | Control: 82.7 (4.7); Test: 68.8 (6.9) |
|  | 72 hours | Mazumder 1996 | 23 | Protein: Mean (and SEM) coefficient of percent nutrient absorption, compared between the control and test groups | Control: 66.9 (4.4); Test: 61.3 (5.8) |
|  | 72 hours | Mazumder 1996 | 23 | Energy: Mean (and SEM) coefficient of percent nutrient absorption, compared between the control and test groups | Control: 82.5 (3.9); Test: 71.8 (5.7) |
|  | 5 days | Rahman 1995 | 66 | Energy: Mean (standard deviation) energy intake (kJ/kg/day) from diet among children with *Shigella* receiving energy-dense study diet compared to 2 control diets. | Control 1: 116 (100); Control 2: 151 (80); Energy-dense: 280 (113). Difference of means (95% CI): Energy-dense vs. control 1 = 113 (46-180); Energy dense vs. control 2: 130 (71-188) |
|  | 3 days | Ramiro Cruz 1994 | 126 | Animal protein: nutrient consumption (% of target) 1-3 days after *Shigella* infection among asymptomatic, dysentery, and diarrhea cases | 62.5% among healthy cases, 48.7% among dysentery cases, and 42.6% among diarrhea cases. |
|  | 3 days | Ramiro Cruz 1994 | 126 | Vegetable protein: Percent nutrient consumption 1-3 days after *Shigella* infection among asymptomatic, dysentery, and diarrhea cases | 75.4% among healthy cases, 61.3% among dysentery cases, and 66.4% among diarrhea cases. |
|  | 3 days | Ramiro Cruz 1994 | 126 | Carbohydrate: Percent nutrient consumption 1-3 days after *Shigella* infection among asymptomatic, dysentery, and diarrhea cases | 93.2% among healthy cases, 80.3% among dysentery cases, and 80.0% among diarrhea cases. |
|  | 3 days | Ramiro Cruz 1994 | 126 | Fiber: Percent nutrient consumption 1-3 days after *Shigella* infection among asymptomatic, dysentery, and diarrhea cases | 91.2% among healthy cases, 53.3% among dysentery cases, and 64.2% among diarrhea cases. |
|  | 3 days | Ramiro Cruz 1994 | 126 | Fat: Percent nutrient consumption 1-3 days after *Shigella* infection among asymptomatic, dysentery, and diarrhea cases | 66.2% among healthy cases, 42.3% among dysentery cases, and 42.1% among diarrhea cases. |
| **Serum retinol** | |  |  |  |  |
|  | Duration of hospitalization | Mitra 1998 | 66 | Mean (SD) serum retinol level (umol/L) at discharge compared to at admission | Discharge: 1.15 (0.50); admission: 0.36 (0.22) |
| **Symptom duration** | | |  |  |  |
|  | Until 48 hours symptom-free | Ballard 2022 | 23 | Mean (SD) duration of vomiting | 2.1 days (4.7) |
|  | Until 48 hours symptom-free | Ballard 2022 | 23 | Mean (SD) duration of fever | 1.7 days (1) |
| **Hospitalization** | |  |  |  |  |
|  | 2 years | Rogawski McQuade 2020 | 755* | Among children who had more than 1 *Shigella*-attributable diarrhea episode, the percent of subsequent episodes with hospitalization | 0% (0.4% in first episode) |
|  | Duration of hospitalization | Mitra 1998 | 66 | Time (days) until discharge (SD) | *S.dysenteriae*: 5.2 (2.2) Other *Shigella*: 3.4 (0.9) |
|  | Duration of hospitalization | Rampengan 1982 | 46 | Average length of treatment/hospitalization | 8.7 days |

Abbreviations: CI, confidence interval; NS, not specified; SD, standard deviation; SEM, standard error of the mean; IQR, inter-quartile range

^a^Scores measured by Bayley-III Scores of Infant and Toddler Development

*Represents # of diarrhea episodes attributable to *Shigella* (rather than number of children with *Shigella*)
